# Quality of conduct and reporting in rapid reviews: an exploration of compliance with PRISMA and AMSTAR guidelines

**DOI:** 10.1186/s13643-016-0258-9

**Published:** 2016-05-10

**Authors:** Shannon E Kelly, David Moher, Tammy J Clifford

**Affiliations:** School of Epidemiology, Public Health and Preventive Medicine, University of Ottawa, H2267A - 40 Ruskin Street, Ottawa, Ontario Canada; Cardiovascular Research Methods Centre, University of Ottawa Heart Institute, Ottawa, Canada; Ottawa Hospital Research Institute, Ottawa, Canada; Canadian Agency for Drugs and Technologies in Health, Ottawa, Canada

**Keywords:** Rapid review, Decision-making, Methodology, Conduct, Quality, Evidence synthesis, Accelerated methods, Reporting, Research transparency, Time factors

## Abstract

**Background:**

Rapid reviews are an accelerated evidence synthesis approach intended to meet the timely needs of decision-makers in healthcare settings. Quality of conduct and reporting has been described in the rapid review literature; however, no formal assessment has been carried out using available instruments. The objective of this study was to explore compliance with conduct and reporting guidelines in rapid reviews published or posted online during 2013 and 2014.

**Methods:**

We performed a comprehensive literature search for rapid reviews using multiple bibliographic databases (e.g. PubMed, MEDLINE, EMBASE, the Cochrane Library) through December 31, 2014. Grey literature was searched thoroughly, and health technology assessment agencies were surveyed to identify additional rapid review products. Candidate reviews were assessed for inclusion using pre-specified eligibility criteria. Detailed data was collected from the included reviews on study and reporting characteristics and variables significant to rapid reviews (e.g. nomenclature, definition). We evaluated the quality of conduct and reporting of included rapid reviews using the A Measurement Tool to Assess Systematic Reviews (AMSTAR) and Preferred Reporting Items for Systematic Reviews and Meta-Analyses (PRISMA) checklists. Compliance with each checklist item was examined, and the sum of adequately reported items was used to describe overall compliance. Rapid reviews were stratified to explore differences in compliance related to publication status. The association between compliance and time to completion or length of publication was explored through univariate regression.

**Results:**

Sixty-six rapid reviews were included. There were heterogeneous nomenclature, research questions and approaches to rapid reviews. Compliance with AMSTAR and PRISMA checklists was poor. Published rapid reviews were compliant with individual PRISMA items more often than unpublished reviews, but no difference was seen in AMSTAR item compliance overall. There was evidence of an association between length of publication and time to completion and the number of adequately reported PRISMA or AMSTAR items.

**Conclusions:**

Transparency and inadequate reporting are significant limitations of rapid reviews. Scientific editors, authors and producing agencies should ensure that the reporting of conduct and findings is accurate and complete. Further research may be warranted to explore reporting and conduct guidelines specific to rapid reviews and how these guidelines may be applied across the spectrum of rapid review approaches.

**Electronic supplementary material:**

The online version of this article (doi:10.1186/s13643-016-0258-9) contains supplementary material, which is available to authorized users.

## Background

Healthcare decision-makers at all levels are under constant pressure to make timely evidence-informed policy or practice decisions. Although highly valued, the time needed to complete a full systematic review of the literature often exceeds the time that end-users have to evaluate evidence or incorporate it into their processes. Rapid reviews are an accelerated evidence synthesis approach specifically intended to meet the needs of knowledge users in healthcare settings [1, 2]. Ideally, to minimize potential sources of bias, a rapid review should follow frameworks for systematic review conduct, such as those published by the Cochrane Collaboration, as closely as time will allow. However, in order to pragmatically achieve timely delivery of evidence, certain concessions are often made in these processes. Attempts have been made to describe and assess rapid reviews through selection and careful appraisal of exemplar samples [1, 3–6]. The characteristics of rapid reviews, and the limitations of these products, have been described in previous work [1, 6, 7]. Heterogeneity of rapid review approaches and poor reporting of methods or processes have been consistently observed, making evaluation of these evidence products difficult [6]. This, in turn, makes it difficult for decision-makers to quantify any bias that may have been introduced or to judge how much value to place on the evidence contained in a rapid review.

Rapid reviews are created by a variety of producers worldwide, including individuals, independent research groups, and organizations and agencies, which offer rapid evidence services to their stakeholders. Many rapid review products are not published, and the majority are not indexed in health-related bibliographic databases (e.g. MEDLINE, CINAHL) [7]. The diverse nomenclature used to describe these approaches also makes it difficult to identify rapid reviews using traditional search methods. This is complicated further by the lack of an accepted or validated definition for rapid reviews, which results in the term ‘rapid review’ having different meanings to the assortment of stakeholders who produce or use them [2, 8].

There are currently no guidelines or accepted rules for the reporting or conduct of rapid reviews. The Preferred Reporting Items of Systematic Reviews and Meta-Analyses (PRISMA) statement and the A Measurement Tool to Assess Systematic Reviews (AMSTAR) checklist are reliable and practical instruments designed to help end-users discriminate between systematic reviews with a focus on quality of reporting and conduct [9, 10]. Both have become widely accepted by publishing agencies and evidence producers since 2011. Given the aim of rapid reviews to optimize to the extent possible a systematic process while synthesizing evidence and balancing the timely requirements of healthcare decision-making, it is feasible that these tools could also be applied to rapid reviews. No studies to date have applied validated reporting or conduct instruments such as PRISMA or AMSTAR to rapid reviews with the goal of assessing the quality of conduct and reporting, although previous work has suggested this task may be helpful to improve reporting transparency [4].

Given the above, and the importance of rapid reviews to decision-makers, this study was carried out to explore the general study characteristics of these research products. We also aimed to evaluate the quality of both process and reporting in both journal-published and unpublished (grey literature) rapid evidence synthesis products through measurement of compliance with the PRISMA and AMSTAR checklists. The secondary aims were to explore whether the time to completion or the length of the report influenced instrument compliance.

## Methods

The strategy for locating rapid reviews and assessing the quality of their conduct and reporting involved three fundamental steps. First, a protocol was developed in August 2011 in consultation with methodological experts in knowledge synthesis, health technology assessment (HTA), and evidence-based decision-making. Second, a broad and comprehensive literature search was carried out to identify published and unpublished samples of rapid reviews produced internationally since 2005. Third, an in-depth examination of the characteristics of the included rapid reviews was conducted. We examined the quality of reporting and process for both published and unpublished rapid reviews using validated tools (AMSTAR and PRISMA) and compared the results to identify areas for improvement. In addition, we explored a variety of common themes identified in previously published work in the area of accelerated evidence synthesis.

### Information sources

Comprehensive literature searches were conducted with the assistance of an experienced medical information specialist knowledgeable in evidence synthesis and rapid reviews. Search strategies were peer-reviewed [11] and used both controlled vocabulary (e.g. National Library of Medicine’s MeSH terms) and keywords. Between October 25 and 31, 2011 we searched PubMed, MEDLINE (Ovid MEDLINE(R) In-Process & Other Non-Indexed Citations, Ovid MEDLINE(R) Daily and Ovid MEDLINE(R) 1948 to Present;), EMBASE (Ovid, 1980 to 2011 Week 42), the Cochrane library, York Centre for Reviews and Dissemination (CRD) Database of Abstracts of Reviews of Effects (DARE), NHS Economic Evaluation Database (EED) and HTA, Web of Science, National Library of Medicine Gateway, and CINAHL (EBSCOHost). Search updates were carried out monthly until December 31, 2014 (see Additional file 1).

A thorough grey literature search was conducted using CADTH’s *Grey matters*: *A search tool for evidence*-*based medicine* (https://www.cadth.ca/resources/finding-evidence/grey-matters-practical-search-tool-evidence-based-medicine) to identify rapid reviews not formally published in peer-reviewed journals and rapid reviews produced by organizations and agencies whose products are not indexed in the bibliographic databases searched. The grey literature search was augmented by a general Internet search (Google/Google Scholar) to identify web-based reports. The searches were supplemented by reviewing the bibliographies of key papers and conference proceedings, citation mapping and hand searching of HTA agencies known to deliver rapid review services. The searches were large in scope and intentionally unrestricted in order to capture the wide variety of published and unpublished products falling under the global term of ‘rapid review’.

### Scan of HTA agency rapid reviews

In order to more comprehensively identify rapid review producers, we supplemented the formal literature search with an email scan of International Network of Agencies for Health Technology Assessment (INAHTA) agencies circulated in November 2011. Each organization was asked:

- Does your agency currently undertake rapid review?
- What timeframes do you offer for your rapid review products?
- If NO, does your agency have plans to produce rapid reviews in the future?
- If YES, what type of rapid review products does your agency produce?
- Are your rapid reviews publicly available on your website? If so, can you please forward the URL/address?

### Screening and selection

Broad eligibility criteria were piloted on a sample of 100 database records following the literature search. These criteria were revised to improve specificity and then applied to each title and abstract identified by one reviewer (LAT) in a standardized manner. A second review author (SK) screened a random sample (10 %) of excluded records. Any uncertainties were resolved by discussion and consensus with a third review author (TC or DM). Any candidate rapid review passing the initial selection criteria with a definite or unclear status, along with all potential samples identified in the grey literature search, was obtained in full-text format. One reviewer (SK) applied the eligibility criteria and made a final decision for inclusion.

### Eligibility criteria

**Fig. 1:**
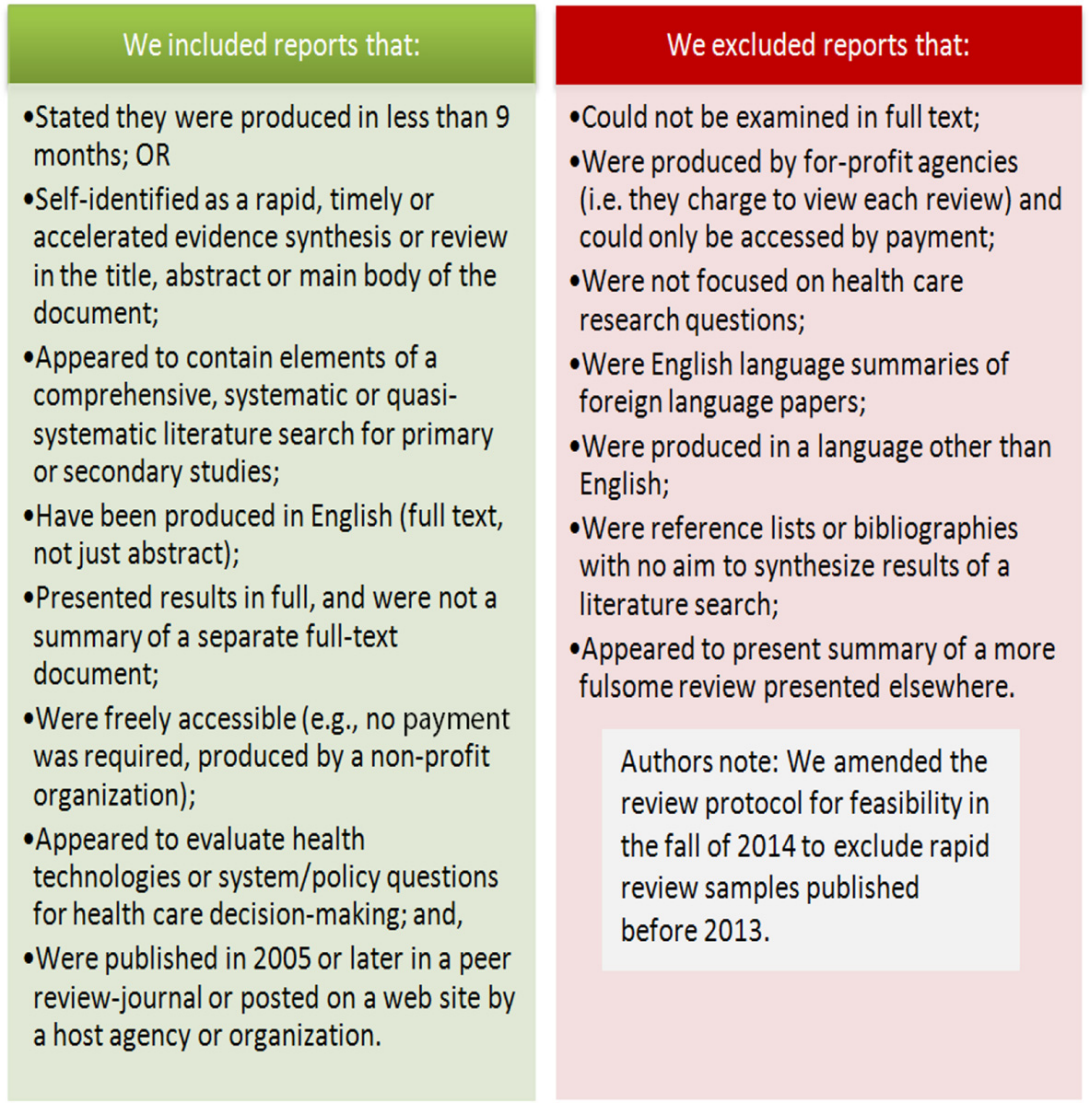
Eligibility criteria for rapid review selection

Due to the diversity of methodologies, production timelines and nomenclature used in the previous rapid review research [1–3], we followed inclusive selection criteria to identify candidate studies (Fig. 1).

Rapid reviews were included regardless of publication status. The protocol was revised prior to data extraction to limit the eligibility of rapid reviews to those produced between January 1, 2013 and December 31, 2014. This revision was necessary in order to obtain a manageable sample of rapid reviews and to ensure that the samples collected reflected current practice given the continuous evolution of rapid review approaches between 2005 and 2014. We included all journal-published rapid reviews meeting the eligibility criteria in the specified timeframe and an equivalent number of agency-produced samples located in the grey literature. Hereafter, journal-published rapid reviews are referred to as ‘published’ while those located in the grey literature are referred to as ‘unpublished’ rapid reviews. Where organizations produced multiple rapid reviews in a single year, a random selection of two samples from all available 2013 or 2014 rapid reviews was made for inclusion. No more than two samples from a single organization were included.

### Data abstraction

A single reviewer (SK) abstracted data into a Microsoft Excel 2007 spreadsheet standardized for the project. Five samples were used to pilot the form, and revisions were made before global extraction. Data was collected from included rapid reviews for study characteristics (e.g. primary author or agency, country, date posted or published, date submitted/accepted (if applicable), number of authors, eligibility and selection criteria, type of research questions, purpose, type of decision under consideration, number of outcomes, funding, use of supplemental appendices), methodological processes (approach, use of protocol/protocol elements reported, number of reviewers screening/extracting/performing assessment of research quality, search date, number of bibliographic databases searched, date/language or geographical filters applied to search or screening, additional search methods employed (e.g. grey literature, hand searching, trial registries, citation mapping) instruments used for quality assessment, use of Grading of Recommendations Assessment, Development and Evaluation (GRADE), types of study included, use of internal or external peer-review), author-reported limitations or disclaimers, rapid review definitions and nomenclature, length of report (in pages) and time frames for completion where reported. We also assessed if findings were framed in context with any reported decision-making need.

In the case where unpublished rapid review methods were not reported or a source document was referenced, agency and organization websites were checked for additional clarification of process. Details on methods or approach were extracted from additional source documents if described in such a way that no variation in process was expected and the process for all rapid review products was standardized and clear. Data were not used if the associated documentation stated that that the method was used ‘sometimes’ or that methods were report-specific.

### Data synthesis and quality appraisal

Data were extracted for published and unpublished rapid reviews separately and then aggregated into a single table for evaluation. Variables were synthesized narratively and summarized using descriptive statistics (frequencies, proportions and percentages) and category groupings (e.g. number of authors, length in pages).

Two dimensions of rapid review reporting and methodological conduct were explored. First, we applied the A Measurement Tool to Assess Systematic Reviews (AMSTAR) checklist, an 11-item measurement tool validated to critically appraise the methodological quality of systematic reviews using currently understood knowledge on bias potentially introduced through conduct in evidence synthesis. We used the AMSTAR checklist to evaluate each included review to examine the overall, and by-item, quality of conduct. Response options for each domain were ‘yes’, ‘no’, ‘can’t answer’ and ‘not applicable’, and domains that were partially answered were recorded by noting which item was answered adequately. We counted each sufficiently reported domain (answer = ‘yes’) and summed responses based on a maximum possible count of 11.

Next, we evaluated the reporting quality of the rapid review samples using the PRISMA statement. The PRISMA statement is a 27-item (and 4-item flow diagram) measure of overall reporting strength for evidence syntheses reporting randomized controlled trials (RCTs). We chose this instrument as it is widely accepted as a scientific standard for reporting of secondary studies of RCTs that can also be applied to other types of research, including healthcare interventions. Response options for each item were ‘yes’, ‘no’ and ‘not applicable’, and we recorded items that were partially answered (e.g. for item 5, if the use of a protocol was mentioned but no registration number was provided). Each included study was evaluated individually, and we counted each sufficiently reported item (answer = ‘yes’) and summed responses based on a maximum possible count of 27.

Overall compliance with PRISMA and AMSTAR were calculated as an overall sum of adequately met items for each rapid review, a mean or median numbers of items reported adequately across all included rapid reviews (overall and by domain), and then stratified by publication status for exploratory analysis.

We explored the potential confounding effect of journal word limits (represented by length in pages) and the impact of time to completion on the number of PRISMA or AMSTAR items adequately reported or met. We carried out univariate regression in Microsoft Office Excel 2007 using the ‘Real Statistics’ data add-in (www.real-statistics.com). Extracted data on the length of the publication (in pages, excluding references and appendices) and reported times to completion for all included studies were used. Rapid reviews were stratified by publication status for additional analyses and documented the proportion of adequately reported PRISMA and AMSTAR checklist items.

## Results

### Selection of rapid review samples

**Fig. 2:**
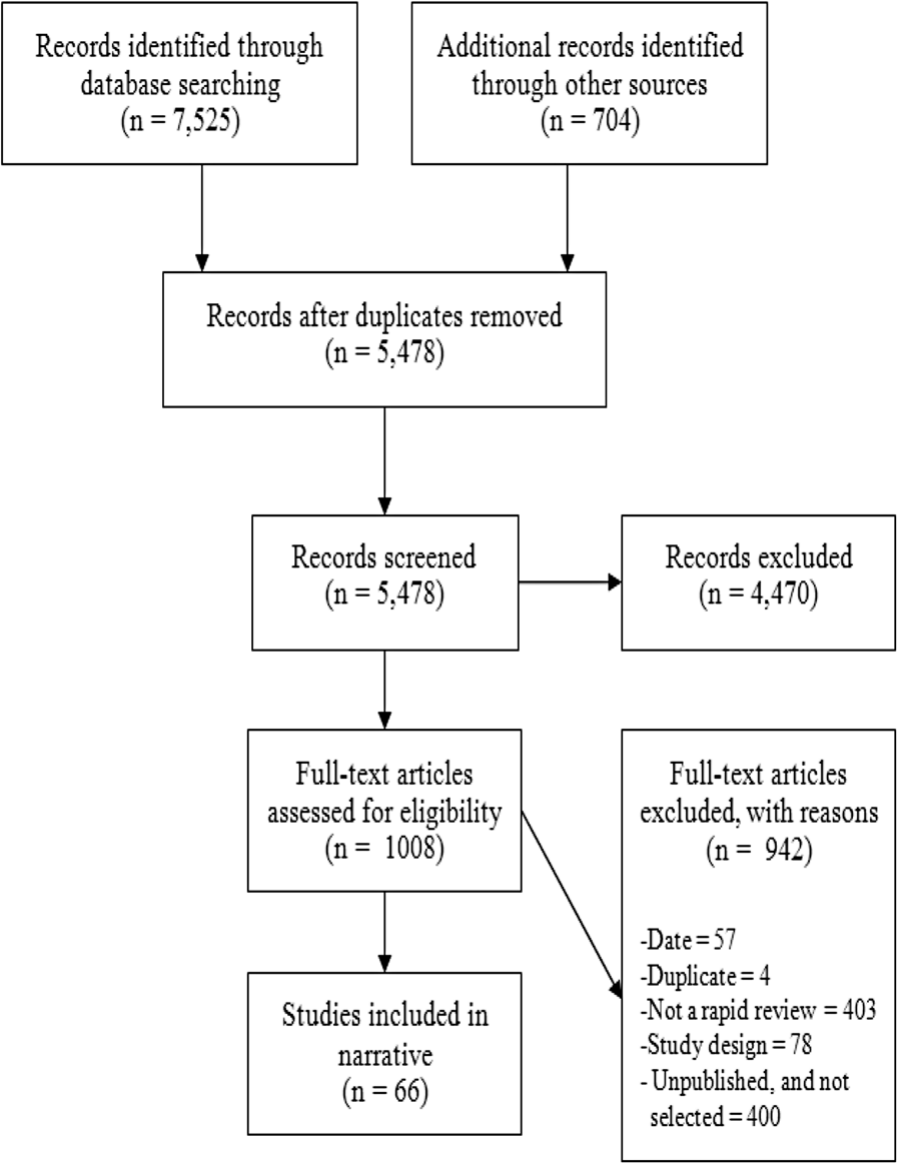
PRISMA flow diagram

Fourteen HTA agencies responded to the INAHTA scan, and their external web sites were searched for relevant rapid review products following the search for published and unpublished reports. The literature search yielded 5478 titles and abstracts after deduplication across databases. In total, 1008 articles were potentially relevant and their full-text was reviewed. Few rapid review samples in the published literature were located prior to 2011; however, samples of unpublished rapid reviews were plentiful. Following full-text review, 66 rapid reviews produced between 2013 and 2014 fulfilled our eligibility criteria and were included [12–77]. Thirty-three were journal-published [12–44] and 33 were unpublished [45–77] rapid reviews. See Additional file 2 for a full list of included and excluded studies. Figure 2 shows a flow diagram of studies included using guidance from the PRISMA statement [78]. Four hundred unpublished rapid reviews met all other eligibility criteria but were not selected with the majority produced by a small number of agencies who author a high volume of rapid reviews per year e.g. (CADTH). The thirty-three published rapid reviews included were reported in 43 published articles together with companion studies [79–86] for one included rapid review that was published in a journal as a series of 10 articles [19]. We considered these publications a single rapid review as they reported results by intervention from a single literature search. Thirty-one unpublished rapid reviews were located on the websites of their producing agency or organization. Two unpublished rapid reviews [72, 76] were located through contact with primary authors following expert input.

### Characteristics of rapid reviews

Table 1 summarizes characteristics of the included rapid reviews. Detailed characteristics could not be reported and explored fully in this study and are reported elsewhere.^1^ Sixty-six rapid reviews were included. Thirty-three were published in 25 unique journals, and the remaining 33 were produced by 31 unique evidence producers, including HTA agencies, academic research groups or international, national or local agencies. The number of rapid reviews published in peer-reviewed journals significantly increased between 2013 and 2014. All published rapid reviews and a majority (88 %) of the included unpublished samples self-identified as a rapid review somewhere in the title, abstract or body of the report. Unpublished studies that did not self-identify as a rapid review were categorized or labelled as a rapid review by their associated agency or organization website through a product description or additional source documentation. The most common countries of production were Canada, USA, the UK and Australia. The number of authors varied greatly amongst the included rapid reviews. Single authorship (a single named individual or attribution to the producing agency only with no individual listing of authors) was found only in the unpublished rapid review samples.

**Table 1:** Characteristics of the included rapid reviews (*n* = 66)

Characteristic	Published (*n* = 33)	Unpublished (*n* = 33)	All (*n* = 66)
Year of production, *n* (%)			
2013	8 (24)	11 (33)^a^	19 (29)
2014	25 (76)	22 (67)^a^	47 (71)
Number of authors, *n* (%)			
1	0 (0)	3 (9)	3 (5)
2–4	14 (42)	11 (33)	25 (38)
5–8	13 (39)	5 (15)	18 (27)
>8	6 (18)	5 (15)	11 (17)
Not reported	0 (0)	9 (12)	9 (14)
Self-identifies as a rapid review, *n* (%)			
Yes	33 (100)	29 (88)	62 (94)
No	0 (0)	4 (12)	4 (6)
Country, *n* (%)			
Canada	5 (15)	13 (39)	18 (27)
USA	4 (12)	3 (9)	7 (11)
UK	11 (33)	9 (27)	20 (31)
Australia	4 (12)	5 (15)	9 (14)
Netherlands	6 (18)	0 (0)	6 (9)
Korea	1 (3)	0 (0)	1 (2)
Switzerland	0 (0)	1 (3)	1 (2)
Malaysia	0 (0)	1 (3)	1 (2)
Various	2 (6)	1 (3)	3 (5)
Rapid review definition, *n* (%)			
Cited	20 (60)	10 (30)	30 (46)
Own	0 (0)	6 (18)	6 (9)
Not reported	13 (40)	17 (52)	30 (46)
Nomenclature, *n* (%)^b^			
Rapid review	14 (15)	10 (30)	24 (36)
Rapid systematic review	9 (12)	1 (3)	10 (15)
Rapid evidence assessment	6 (18)	4 (12)	10 (15)
Rapid evidence synthesis	2 (6)	1 (3)	3 (5)
Rapid synthesis	0 (0)	1 (3)	1 (2)
Rapid review of systematic reviews	1 (3)	1 (3)	2 (3)
Systematic rapid evidence assessment	1 (3)	0 (0)	1 (2)
Evidence-based analysis	0 (0)	1 (3)	1 (2)
Rapid response	0 (0)	2 (6)	2 (3)
Rapid evidence report/review	0 (0)	6 (18)	6 (9)
Evidence briefing	0 (0)	1 (3)	1 (2)
Evidence map	0 (0)	1 (3)	1 (2)
Rapid advice guideline	0 (0)	1 (3)	1 (2)
Systematic rapid evidence review	0 (0)	1 (3)	1 (2)
None used	0 (0)	1 (3)	1 (2)
Research question^c^, *n* (%)			
Clinical efficacy	18 (55)	22 (67)	40 (61)
Clinical effectiveness	16 (48)	25 (76)	41 (62)
Safety	13 (30)	15 (45)	28 (42)
Diagnostic/screening test	2 (6)	1 (3)	3 (5)
Health economics/cost	4 (12)	14 (42)	18 (27)
Guidelines	1 (3)	7 (21)	8 (12)
Public health	6 (18)	5 (15)	11 (17)
Health systems	9 (27)	11 (33)	20 (30)
Health policy	5 (15)	3 (9)	8 (12)
Service delivery	9 (27)	12 (36)	21 (32)
Other^d^	5 (15)	5 (15)	10 (15)
Synthesis method, *n* (%)			
Narrative	31 (94)	27 (82)	58 (88)
Meta-analysis	2 (6)	2 (6)	4 (6)
Indirect comparison	0 (0)	1 (3)	3 (5)
Economic evaluation	0 (0)^e^	0 (0)	0 (0)
None (no studies located)	0 (0)	3 (9)	3 (5)
Length of publication, number of pages^f^, *n* (%)			
1–5	5 (15)	3 (9)	8 (12)
6–10	17 (52)	6 (18)	23 (35)
11–15	9 (27)^g^	3 (9)	12 (18)
16–20	2 (6)	5 (15)	7 (11)
20–50	0 (0)	10 (30)	10 (15)
>50	0 (0)	6 (18)	6 (9)
Length of publication, mean (SD)	8.8 (4.03)	22.8 (27.2)	18.7 (21.7)

*RR* rapid review, *SD* standard deviation

^a^Proportion matched by year and limited in number by those published, proportion does not reflect the actual number of unpublished rapid reviews

^b^For unpublished refers to the terminology used to describe the methodology employed, not the product name assigned by the organization. Some publication identified by multiple names, but this data reflects the most commonly used term in the publication

^c^Multiple research questions per rapid review

^d^Quality indicators, epidemiological associations, healthcare study methodology, patient experience

^e^A single study in the published group did a narrative of economic evaluations, other simply analysed costs reported. No study did a de novo economic evaluation

^f^Without references or appendices, including figures. One unpublished report was a webpage only and was counted as five pages approximated to its content. Results sum the number and percentage of rapid reviews in each page range

^g^Mean across 10 included multiple publications for the same RR used

Nomenclature used to describe the accelerated or timely evidence synthesis process varied greatly amongst the included studies. The terms ‘rapid review’ (36 %), ‘rapid systematic review’ (15 %) or ‘rapid evidence assessment’ (15 %) were most common. Over 60 % of the research questions were aimed at the clinical efficacy or effectiveness of an intervention. Health economics, cost questions and those related to healthcare systems or service delivery were also frequent. Few samples addressed questions related to diagnostic or screening tests. A large proportion (88 %) of the included studies narratively summarized results.

Twelve [17, 18, 26, 29, 32–34, 50, 64, 74, 76] studies considered meta-analysis, but data was insufficient for pooling which necessitated a narrative summary of results. Four [17, 26, 64, 74] rapid reviews conducted meta-analysis, and a single review conducted an indirect treatment comparison [76]. None of the included studies reported mentioned PRISMA or AMSTAR guidelines in their report, although one study did report using PRISMA-P guidelines for their protocol [76]. Included rapid reviews had a mean length in pages of 18.7 (standard deviation (SD) = 21.7) without considering references and appendices. Twelve percent (*n* = 3) of the journals publishing the included rapid reviews required PRISMA in their instructions to authors. We were unable to ascertain if any of the agencies or groups producing unpublished rapid reviews endorsed PRISMA or AMSTAR use.

### Length of time taken to complete a rapid review

Although 98 % of our included samples used language describing rapid, accelerated or timely conduct and reporting of an evidence synthesis, very few reported how long it took to carry out the review. Three of the published rapid reviews [12, 29, 33] reported time to completion of 6 weeks (*n* = 2) or 8 weeks (*n* = 1). Eight of the unpublished rapid reviews reported actual time to completion [47, 48, 58–60, 66, 70, 77] of between 3 and 18 weeks (mean 9.9, SD 4.8).

In published samples that did not report time to completion, we estimated duration in weeks through the use of the date of the literature search and calculated the number of days before the review was submitted to a journal. In 21 samples that reported both a date for the literature search and for journal submission, the mean time to completion was 36.3 weeks (SD 25.8).

### Methodological quality of rapid reviews: compliance with the AMSTAR checklist

**Fig. 3:**
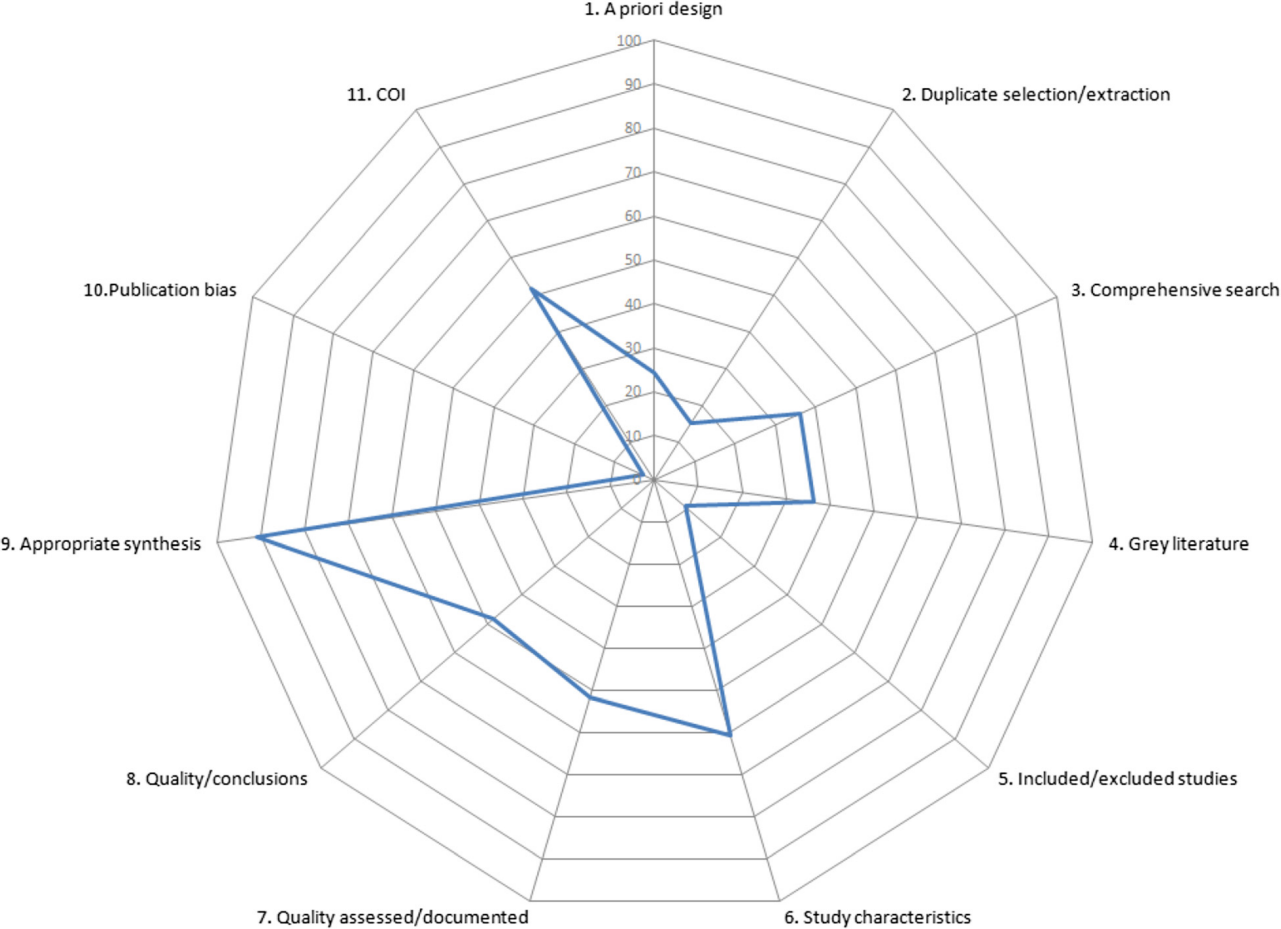
Star chart depicting proportions of rapid reviews adequately reporting AMSTAR items (*n* = 66). *COI* conflict of interest

Figure 3 shows the proportion of rapid reviews (*n* = 66) that adequately met the individual AMSTAR checklist domains. Overall, compliance with the 11 items was poor. The median number of AMSTAR domains fulfilled was 4 (interquartile range (IQR) = 2.5 to 6.0) out of the maximum possible 11 items. Domains were adequately met 39 % of the time, on average, in the 66 included rapid review samples.

Items that were better reported than others were the appropriateness of the methods used to combine the findings of studies (item 9, 91 %), the aggregated study characteristics (item 6, 61 %), assessment and documentation of study quality (item 7, 52 %), appropriately forming conclusions based on the quality of the included studies (item 8, 48 %) and listing study sources of funding (item 11, 52 %). Compliance was extremely poor for the inclusion of a priori design of the research question(s) and inclusion criteria (item 1, 24 %), duplicate study selection and extraction (item 2, 15 %), use of the publication status as an inclusion criterion (item 4, 36 %) and providing a list of included and excluded studies (item 5, 9 %). Further exploration of item 2 (duplicate study selection and extraction) showed that 23 % of rapid reviews limited either study selection or data extraction to a single reviewer (with or without checking by a second reviewer) and only partially met this domain. Seventy-four percent of rapid reviews partially met item 5 by providing references for included studies but not excluded studies. Only two rapid reviews [58, 59] reported any formal assessment of publication bias, and none presented any graphical aids (e.g. funnel plot) to support their evaluation.

### Variables associated with AMSTAR reporting

Exploratory univariate regression for AMSTAR could not be carried out for any variable as the data for the 66 included rapid review samples did not satisfy the normality assumption according to a Shapiro-Wilk test. Square root and logarithmic data transformations were attempted but did not normalize the distribution.

The smaller set of 11 rapid reviews reporting time to completion was normally distributed according to a Shapiro-Wilk test. Results of the exploratory regression on this variable showed that longer time to completion was significantly associated with an increase in the number of AMSTAR domains met (regression coefficient 1.7, 95 % confidence interval (CI) 0.2 to 3.2).

### Reporting of rapid reviews stratified by publication status: compliance with the AMSTAR checklist

**Fig. 4:**
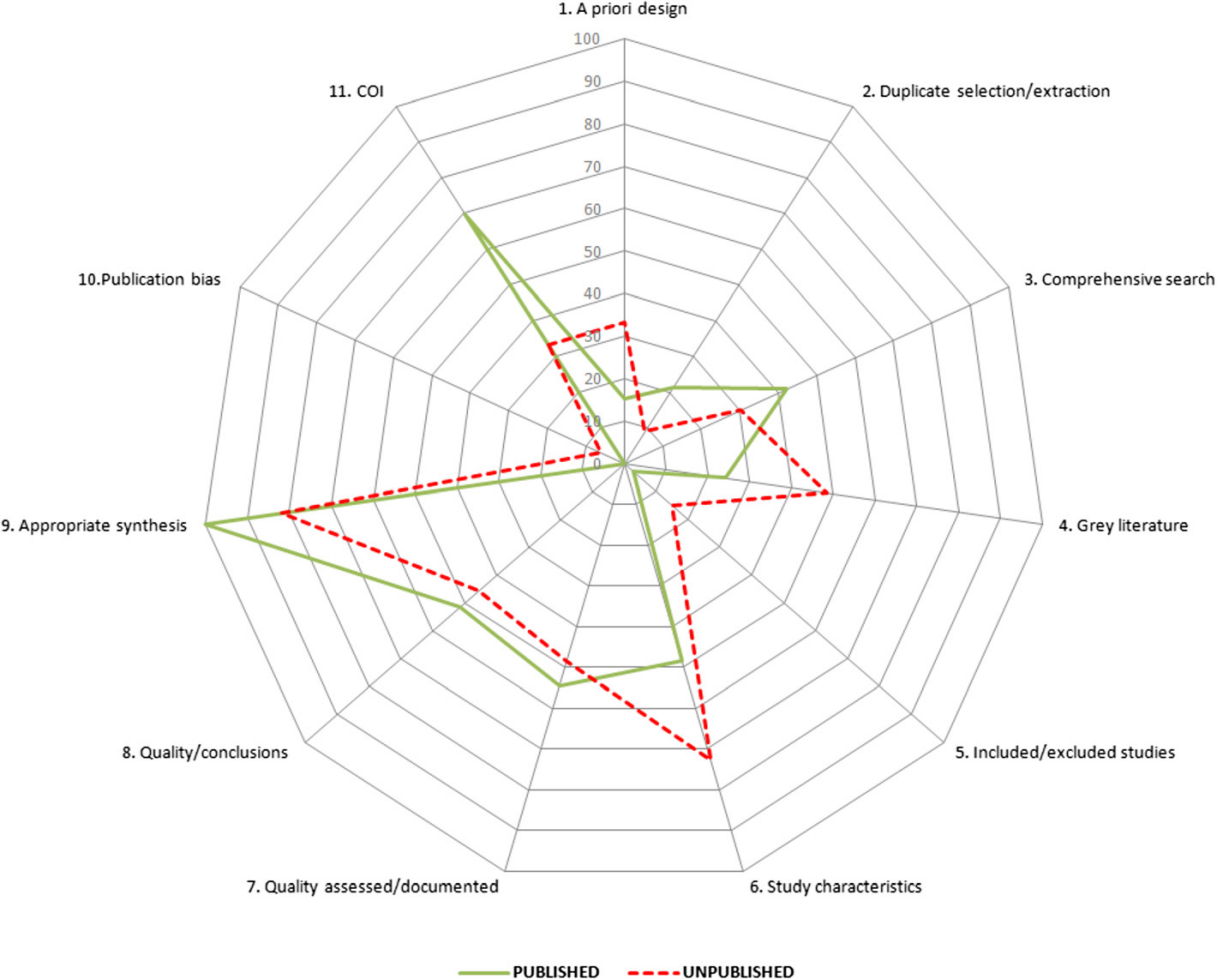
Star chart depicting proportions of rapid reviews adequately reporting AMSTAR checklist items, by publication status (*n* = 66: *n* = 33 published, *n* = 33 unpublished). *COI* conflict of interest

The mean number of AMSTAR domains adequately met for published rapid reviews was 4.2 (SD 2.2) out of the maximum possible of 11 and 4.3 when unpublished (SD 2.5). Table 2 reports the proportion of published and unpublished rapid reviews meeting individual AMSTAR domain specifications (answer = ‘yes’). A higher proportion of unpublished rapid reviews provided an a priori design (item 1) and searched for reports regardless of their publication type (grey literature, item 4) when compared to published reviews, although none of the 66 included rapid reviews reported this more than one third of the time. Higher proportions of published rapid reviews met the AMSTAR requirements for appropriately combining the findings of studies (item 9) and for declaring sources of support through conflict of interest statements (item 11). Poor reporting of excluded studies led to extremely low number of ‘yes’ responses for item 5 in all rapid reviews, and similar proportions of rapid reviews met AMSTAR domain requirements for the reporting of literature searches (Fig. 4). Many rapid reviews received ‘partial’ responses for this domain as two or more databases were searched in a large proportion of rapid reviews; however, they did not employ supplementary strategies, or methods were so restricted (e.g. searched only 2 years of literature) that the strategy could not be considered comprehensive to fulfil this domain requirement in the studies evaluated.

**Table 2:** Comparison of compliance to conduct standards outlined by AMSTAR

Item	Published (*n* = 33) (%)	Unpublished (*n* = 33) (%)
1. Was an ‘a priori’ design provided?	15.2	33.3
2. Was there duplicate study selection and data extraction?	21.2	9.1
3. Was a comprehensive literature search performed?	42.4	30.3
4. Was the status of publication (i.e. grey literature) used as an inclusion criterion?	24.2	48.5
5. Was a list of studies (included and excluded) provided?	3.0	15.2
6. Were the characteristics of the included studies provided?	48.5	72.7
7. Was the scientific quality of the included studies assessed and documented?	54.5	48.5
8. Was the scientific quality of the included studies used appropriately in formulating conclusions?	51.5	45.5
9. Were the methods used to combine the findings of studies appropriate?	100	81.8
10. Was the likelihood of publication bias assessed?	0.0	6.1
11. Was the conflict of interest included?	69.7	33.3

### Variables associated with AMSTAR reporting, stratified by publication status

Results from a univariate regression on the length of publication (in pages) showed no association with number of overall AMSTAR items fulfilled when published rapid reviews were analysed. Data for the unpublished rapid review samples were normalized using a square root transformation prior to analysis. Regression coefficients showed a significant association (regression coefficient 6.2 (95 % CI 2.99 to 9.43)) between the length of report and the total number of AMSTAR items met. The sample size of rapid reviews reporting time to completion (*n* = 11) was insufficient for regression analyses.

### Reporting of rapid reviews: compliance with the PRISMA statement

**Fig. 5:**
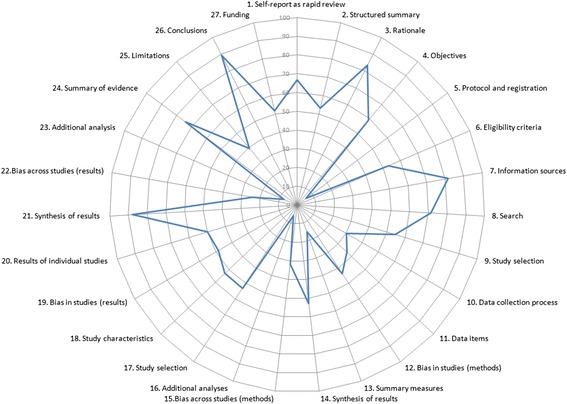
Star chart depicting proportions of rapid reviews meeting PRISMA reporting guidelines by item (*n* = 66)

The mean number of adequately reported PRISMA items was 13.2 (SD 6.0) out of the maximum possible 27. Items were adequately reported 49 % of the time on average in the 66 included rapid review samples. Figure 5 shows the proportion of rapid reviews that adequately reported the individual PRISMA checklist items. Individual items that were reported well in a large proportion of rapid reviews were the following: describing all information sources in the search (item 7, 81 %), presenting the main results of the review in a synthesis of results (item 21, 88 %), summarizing the main findings with relevance to key groups (item 24, 74 %) and providing general interpretation and context for the results of the review in the conclusions (item 26, 89 %). Other items were very poorly reported, such as indicating if a protocol exists or is registered (item 5, 6 %), describing the process of data collection (item 10, 30 %) and discussing the study limitations at the study/outcome and review level (item 25, 40 %). Less than 50 % of rapid reviews described the methods used for assessing risk of bias in individual studies (item 12, 44 %) or presented data on the risk of bias in each study (item 19, 48 %).

Due to the narrative description of results in most of the included rapid review samples, items 16 (additional analyses—methods, 6 %) and 23 (additional analyses—results, 8 %) were often given responses of ‘not applicable’ in the rapid review samples. Summary measures (item 13) were also poorly reported in a large proportion of included studies. Of the 14 rapid reviews who stated their intention to carry out meta-analyses in their methods, only 4 (29 %) reported the effect measure that would be considered in their analyses. Three included unpublished rapid reviews were ‘empty’, meaning that no candidate studies met the eligibility requirements.

A total number of PRISMA items reported by rapid review were used in a subsequent exploratory regression as the data were normally distributed according to a Shapiro-Wilk test (with some negative skewness and kurtosis).

### Variables associated with PRISMA reporting

Exploratory regression analyses were carried out for the total number of PRISMA items adequately reported using report length and time to completion as variables. The length of the primary publication in pages (without appendices or references) was associated with an increase of 1.45 PRISMA items adequately reported (95 % CI 0.6 to 2.3). A smaller set of rapid reviews (*n* = 11) reporting time to completion did not find a significant correlation between the number of weeks required to complete a rapid review and the number of PRISMA checklist items adequately reported (regression coefficient 0.23 (95 % CI −0.27 to 0.72)).

### Reporting of rapid reviews stratified by publication status: compliance with the PRISMA statement

**Fig. 6:**
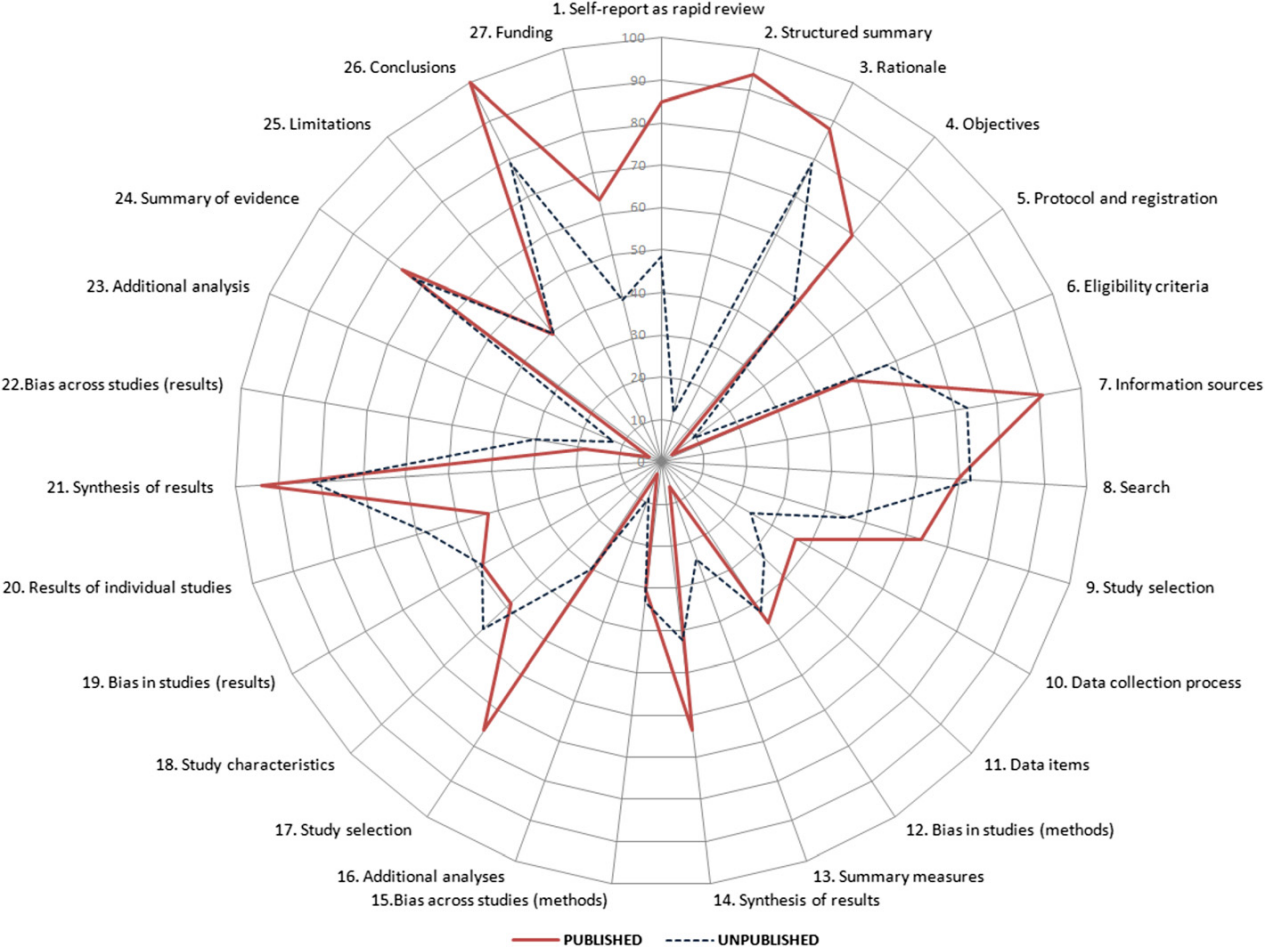
Star chart depicting proportions of rapid reviews adequately reporting PRISMA items, by publication status (*n* = 66: *n* = 33 published, *n* = 33 unpublished)

The mean number of adequately reported PRISMA items for published rapid reviews was 14.5 (SD 4.7) out of the maximum possible 27 and 11.7 when unpublished (SD 6.8). Items were adequately reported 53 % of the time, on average, in the 33 included published rapid review samples and 44 % of the time in unpublished samples. Table 3 shows the results of a comparison of total number rapid reviews adequately reporting PRISMA items, stratified by publication status. PRISMA items are better reported in published rapid reviews for the five categories: identifying the report as a rapid or accelerated evidence synthesis (a slight modification to the item for the purposes of this review) in the title (item 1), using a structured abstract (without protocol registration number considered) (item 2), stating the selection process for inclusion of studies (item 9), providing a general interpretation of results in the form of conclusions (item 26) and declaring sources of funding for the rapid review (item 27). Published rapid reviews more often described information sources, syntheses of results, results of the study selection process and synthesis of results (Fig. 6). Unpublished rapid reviews were more likely to clearly state eligibility criteria (item 6) for article selection and present a full electronic search strategy (item 8) and study characteristics (item 18). Rapid reviews reported study rationale, risk of bias methods and results, synthesis of results and summaries of evidence equally, regardless of publication status.

**Table 3:** Comparison of compliance to PRISMA reporting guidelines

PRISMA item	Published (*n* = 33) (%)	Unpublished (*n* = 33) (%)
Title		
1. Self-reports as a rapid review	84.85	48.48
Abstract		
2. Structured summary	93.94	12.12
Introduction		
3. Rationale	87.88	78.79
4. Objectives	69.70	48.48
Methods		
5. Protocol and registration	3.03	9.09
6. Eligibility criteria	48.48	57.58
7. Information sources	90.91	72.73
8. Search	69.70	72.73
9. Study selection	63.64	45.45
10. Data collection process	36.36	24.24
11. Data items	39.39	33.33
12. ROB in individual studies	45.45	42.42
13. Summary measures	25.00*	50.00*
14. Synthesis of results	63.64	42.42
15. ROB across studies	30.30	33.33
16. Additional analyses	3.03	9.09
Results		
17. Study selection	75.76	30.30
18. Study characteristics	48.48	57.58
19. ROB within studies	48.48	48.48
20. Results of individual studies	42.42	57.58
21. Synthesis of results	93.94	81.82
22. ROB across studies	18.18	30.30
23. Additional analysis	3.03	12.12
Discussion		
24. Summary of evidence	75.76	72.73
25. Limitations	39.39	39.39
26. Conclusions	100.0	78.79
Funding		
27. Funding	64.64	39.39

*Indicates that statistic was calculated only for rapid reviews that aimed to perform statistical synthesis/meta-analysis

### Variables associated with PRISMA reporting, stratified by publication status

A number of PRISMA items adequately reported were analysed in a subsequent exploratory regression stratified by publication status as the data were normally distributed according to a Shapiro-Wilk test. Separate univariate regression analyses were carried out for the PRISMA item compliance using the length of report and time to completion as variables, stratified by publication status. Results for the published rapid review samples showed that the length of the primary publication was associated with a significant increase in PRISMA items adequately reported (regression coefficient 0.4, 95 % CI 0.1–0.6). The length of publication in the unpublished samples was associated with a larger increase in adequately reported PRISMA items (regression coefficient 2.5, 95 % CI 1.3–3.6).

## Discussion

Our study provides a comprehensive assessment of the design and reporting characteristics of a large, recent cohort of rapid reviews. A detailed examination of baseline characteristics showed a heterogeneous mix of nomenclature, research questions and approaches to rapid reviews from a relatively small number of countries. The rapid review samples showed poor compliance with both the PRISMA and AMSTAR checklists. Only selected items in both instruments were adequately addressed by any rapid review. Stratification by publication status revealed that a higher proportion of published rapid reviews adequately complied with PRISMA guidelines compared to the unpublished samples. There was no difference in the number of AMSTAR domains met when publication status was considered. There was evidence of an association between the length of publication (PRISMA) and time to completion (AMSTAR) on the total number of items reported or met in this cohort of rapid reviews. This relationship with the length of report was reciprocated when the rapid reviews were stratified by publication status. To our knowledge, this is the first study to explore the compliance of rapid reviews with the PRISMA and AMSTAR instruments or any other standardized tool that captures adequacy of reporting and conduct. No other publications have aimed to study published rapid reviews or make comparisons taking publication status into consideration.

This research follows previous studies examining the transparency of process and quality of reporting in rapid reviews without the use of standardized tools or instruments. In line with the results of previous work, many of the identified studies did not report methods in sufficient detail [1, 3, 5], a problem not unique to rapid reviews [87]. Although there were differences in the number of PRISMA and AMSTAR items met when published and unpublished reviews were considered separately, the limited details reported in the rapid reviews prevented a comprehensive evaluation in some domains, notably literature search, quality assessment, screening, selection and extraction, all of which are important contributors to the reproducibility and evaluation of a review. Any or all of these components may be tailored or even omitted as part of the streamlining processes designed to expedite systematic review completion times [3] so were unable to ascertain whether the reporting omissions were attributable to the rapid review approach or simply poor reporting [88]. Evaluations of reporting characteristics in systematic reviews and meta-analyses have noted similar shortcomings as were found in this study, especially in non-Cochrane reviews [88–90].

Research protocols are a key feature of systematic reviews, and their absence leaves rapid reviews open to selective reporting of results or conclusions and facilitates post hoc modifications of approach [91]. Our findings show that few rapid review authors are reporting the use of protocols in their publications and even fewer are registering or publishing them. This is reflected in the poor compliance with related items in both the PRISMA and AMSTAR checklists. Our results conflict with a recent report by Polisena et al. who reported a cross-sectional review of processes and methods based on a survey of 29 international rapid review programmes [4]. Their results showed that 96.6 % (*n* = 28) of the agencies queried incorporated protocol development into their rapid review process. Many of the rapid reviews included in this study were produced by the agencies and organizations in the Polisena survey. There are a number of reasons that authors or organizations may choose not register their rapid review protocols, including the belief that systems like PROSPERO are for systematic reviews only, which emphasizes the viewpoint that a rapid review is not a systematic review [7]. Authors of rapid reviews may use a more condensed version of a protocol to ensure a timely start to the review process, but we were unable to assess this in our samples. Rapid reviews also show variety in the types of study questions they answer, including public health, health services and system questions. Applicability of PROSPERO or Preferred Reporting Items for Systematic Review and Meta-Analysis for protocols (PRISMA-P) guidance outside of the realm of therapeutic efficacy may be questioned [92], although they may still provide useful guidance to authors of rapid reviews [93].

It is concerning that the reporting of limitations specific to the conduct of the rapid review was consistently poor given the inherent methodological tailoring which motivates the approach; however, this finding is consistent with previous work. Ganann et al. also found that very few rapid reviews discussed potential limitations or any potential bias that may have been introduced due to methodological concessions [1]. Some rapid reviews in this study reported limitations, but a large proportion highlighted issues related to the quality of the included studies rather than weaknesses in approach or conduct. Disclaimers were used, most often in unpublished reports, to highlight caveats to end-users; however, most focused on the currency of the review or vaguely referenced that the review could not be considered comprehensive. It is particularly important for rapid reviews to have a comprehensive limitation section to enable the end-user to judge the validity of the methods employed, the studies included and ultimately how applicable the findings of the review may be for their own purposes.

Less than half of rapid reviews reported a structured summary or abstract. When the rapid review samples were stratified by their publication status, results changed significantly. We found that almost all journal-published samples reported structured abstracts, as is usually required by the journal, while few unpublished samples did. Many of the unpublished rapid reviews did use executive summaries to highlight key findings; however, most were either too lengthy to provide the reader with the intended brief overview of the report and did not summarize methods in any capacity. This may be attributable to the different aim of unpublished rapid reviews, which may be presented to end-users alongside additional support documentation (e.g. research summary document). It is probable that these rapid reviews are not aimed at being a stand-alone product like a journal article [94]. Still, the Organization for Economic Co-operation and Development (OECD) and others still highlight the need for key messages over and beyond the executive summary as they often cannot read detailed the findings from HTA products and must have critical messages communicated for better knowledge translation and uptake [94, 95].

Although closely related, it is important to keep the concepts of reporting and conduct separate when considering these findings. The low number of items completely met, and large proportion of domains of partially met for AMSTAR may be attributable to more stringent or rigid assessment requirements (when compared to items in PRISMA). For example, the use of single reviewers to screen literature or extract data resulted in low number of AMSTAR items adequately meeting related domains. It is possible that a more granular AMSTAR assessment criterion would capture the methodological tailoring intrinsic to rapid reviews in a way that is more descriptive to the end-user. Further detail on the individual methods tailored in a rapid review, if captured by AMSTAR, would allow for more careful consideration of what is acceptable or not and where bias, if any, was likely to be introduced. It may be useful to have subdomains for item 1 where the use of a protocol would be kept separate from the description of eligibility criteria or the PICO, item 2 (duplicate study selection and data extraction) or item 5 (list of included and excluded studies). While not aimed at rapid reviews, a modification to the AMSTAR checklist in the form of R (revised)-AMSTAR has been proposed and validated [96] but was not developed or endorsed by the AMSTAR research group. R-AMSTAR considers three to four more detailed criteria per original AMSTAR item, which in principle may be more applicable to assessment of rapid reviews. This tool was later assessed to have poor measurement properties compared to the original instrument in a sample of systematic reviews, so further research or revision may be required before investigation of its use can be considered [96, 97].

Regression models were used to explore the influence of article length and completion time on the number of PRISMA and AMSTAR items adequately reported or met. Longer manuscript length was associated with higher compliance with PRISMA guidelines while AMSTAR compliance increased with time to completion. No previous studies have assessed rapid reviews using these methods, so there is little evidence to aid with interpretation of the significance of this result. Findings suggest reporting quality is compromised when not enough space is provided for a fulsome description. Results from a study on the reporting of meta-analyses of surgical interventions had similar findings [89] and demonstrate that this is not a problem specific to rapid reviews alone.

The term ‘rapid review’ implies that time is a significant factor in the conduct and reporting of this evidence synthesis approach. The influence of time was significant in our regression, and rapid reviews with longer time to completion (in weeks) showed a higher proportion of PRISMA and AMSTAR items adequately reported or met. Harker and Kleijnen examined a sample of rapid reviews and estimated time taken to complete based on the last search date and date published [3]. Their results showed a significant association between the length of time taken and the number of rapid review methodologies that reported clearly and closely adhered to recommended systematic review guidelines from Cochrane or the York Centre for Reviews and Dissemination (CRD). This empirical evidence may actually conflict with the stance of researchers and knowledge users on this topic. In a recent study on research producer and healthcare decision-maker opinions and attitudes towards rapid reviews, Kelly et al. (2015, under review) noted there is a salient viewpoint amongst this mixed group of evidence producers and knowledge users that reduced review time is not necessarily associated with a lower quality of review and they asserted that quality can be maintained even when rigour is reduced. Our analyses did not take into account the differences in rapid review approach. It may be more sensible to consider reporting and conduct of rapid reviews while also examining the variation in review approach (from an evidence brief or map to a fully comprehensive report that resembles a systematic review in almost all facets) which may also influence time to completion. Data were insufficient to explore these factors through multiple regression, but this may be a valuable research objective going forward.

### Strengths and limitations

The strengths of this research include a protocol-driven design (available on request through the corresponding author), a complete and far ranging search and a detailed exploration into the reporting and conduct of rapid reviews.

Limitations of this study may influence the interpretation and applicability of our findings. Due to the diversity of approaches, timelines and nomenclature employed by rapid review producers, it was difficult to apply a standardized selection criteria and some subjectivity by the reviewers may have influenced inclusion of candidate rapid reviews. Specifically, the eligibility criterion of 9 months for the time to completion of rapid reviews was difficult to apply; therefore, most reviews met the inclusion criteria by self-identifying as an accelerated, rapid or timely review of evidence in the title, abstract or full-text. As we were unable to corroborate time to completion in most articles, we cannot exclude that some producers may have undertaken comprehensive systematic reviews (with little tailoring of methods) in an accelerated fashion using additional resources. Although Moher et al. acknowledge that this approach may still be a type of rapid review, the inclusion of these comprehensive but timely reports may skew the study of the tailored methods [93].

Similarly, the noted heterogeneity of terminology may have led to some rapid reviews being missed. Although we limited the rapid reviews we retrieved to a cross section of samples from 2013 and 2014, we believe we captured a comprehensive representation of reviews in both the published and grey literature. The addition of the INAHTA scan also supplemented our search and identified smaller organizations producing rapid reviews that helped add to our unpublished samples. Additionally, when the protocol was amended to restrict the date of publication for the included rapid reviews to 2013 and 2014, some rapid review producers were omitted from the sample, having not published rapid reviews within our eligibility timeline. This necessitated the inclusion of two rapid review samples each from two HTA producers (CADTH, Health Quality Ontario).

In this study, we explored two influential variables and their association with the PRISMA and AMSTAR checklists through univariate regression. Further exploration of other influential variables is warranted including the inclusion of primary or secondary studies (or both), type of research questions, purpose or type of rapid review approach or journal factors such as PRISMA endorsement or impact factor. In addition, looking at the total aggregate number of items adequately reported or met for both instruments as absolute measures of compliance is a weakness. These summary totals have limited utility to end-users, as there is currently no accepted cut-off denoting low, moderate or high quality of conduct or reporting based on these totals. We have described individual domain compliance in an effort to make results more practical and to facilitate interpretation in the context of rapid reviews. These instruments were designed for use with systematic reviews and meta-analyses of therapeutic interventions, and it could be argued that measuring our sample rapid reviews involving a variety of research question types on the same scale is unfair. However, there are limited studies comparing the methods of rapid reviews to those of more fulsome systematic reviews [5, 98–100]. Instruments like PRISMA or AMSTAR enable comparisons of rapid reviews in a standardized manner to each other. This could be extended to allow for comparisons against other evidence synthesis products. These tools provide evidence producers with a concise, consistent way to ensure that reviews are transparently and adequately reported to end-users, which enables decision-makers to assess findings they receive with confidence, especially when they must do so urgently when decisions cannot wait.

We included three ‘empty’ rapid reviews, where no studies of interest were eligible for inclusion. There is no clear-cut way to assess these reviews, and although some have suggested these studies should be excluded, we elected to include them [101]. Although AMSTAR and PRISMA compliance was low in these samples, these empty reviews are valuable to end-users as they highlight knowledge gaps, communicate topics to reduce duplication and indicate that state of evidence at a certain point of time, which is often the primary objective of a rapid review [102]. Care should be taken to prevent publication bias [103]. The authors of the empty rapid review samples in this study still provided conclusions based on a broader review of relevant literature but often failed to highlight these shortcomings to the end-user. In many of the other included rapid reviews, eligibility criteria were expanded to consider more evidence when no studies of a certain type were located, an iterative process that is common in both systematic and rapid reviews [2, 9]. Without a protocol that is posted or registered, it is difficult to assess whether these modifications were appropriate, even when the processes are well-described. Additionally, a series of 10 rapid reviews on chronic pain that used a single search were included as a single rapid review with accompanying companion articles. Although it would not have changed the results of this study, it is worth mentioning that employing a single search for multiple evidence reviews may be an approach used to expedite the process, and it may have been more appropriate to consider these reviews individually.

## Conclusions

Rapid review products vary greatly, and it is complicated and onerous to compare and contrast the numerous approaches used. Standardized reporting and conduct checklists such as AMSTAR and PRISMA provide a useful way to compare and contrast rapid reviews across a number of key domains. This assessment of 66 rapid reviews shows that conduct and reporting are often inadequate and unclear. This is a significant limitation attributable to rapid reviews, although this problem is not unique to this approach. Poor descriptions of research activities result in research that is not replicable and end-users are unable to sufficiently assess potential for bias in the reports. Arguably, clear and complete descriptions of conduct and transparent reporting of research activities are more important in rapid reviews as they inherently tailor gold standard review methodology. Editorial insistence may help to encourage compliance in published reports; however, scientific editors, authors and producing agencies all have a responsibility to ensure that the reporting of conduct and findings in the research products they produce or publish is appropriate and complete. Our results show that future research may be warranted to define reporting or conduct guidelines specific to rapid reviews. This is not to imply that existing guidelines are inadequate, but further work to evaluate their adequacy for assessing rapid reviews may be required. It is unclear whether guidelines specific to rapid reviews are necessary or desired by the evidence producers and users and whether these types of products would be applicable across the spectrum of rapid review approaches.

## Additional files

Additional file 1: Search Strategy. Search Strategy and MeSH/keywords used for searching. (DOCX 32 kb) Additional file 2: Included and Excluded Study Lists. Details the sample of rapid reviews included in this manuscript and reports excluded studies. (DOCX 27 kb)

## Abbreviations

AMSTAR: A Measurement Tool to Assess Systematic Reviews
CI: confidence interval
CRD: York Centre for Reviews and Dissemination
GRADE: Grading of Recommendations Assessment, Development and Evaluation
HTA: health technology assessment
INAHTA: International Network of Agencies for Health Technology Assessment
IQR: interquartile range
OECD: Organization for Economic Co-operation and Development
PRISMA: Preferred Reporting Items of Systematic Reviews and Meta-Analyses
RCT: randomized controlled trial
SD: standard deviation

## Footnotes

1 Note: The rapid review characteristics presented in this study are free from selective reporting. The surplus of study and methodological characteristics could not be adequately addressed in a single manuscript and will be explored in a future manuscript.
