# Supplementary material for: Quality of conduct and reporting in rapid reviews: an exploration of compliance with PRISMA and AMSTAR guidelines
Source: Syst Rev. 2016 May 10;5:79. doi: 10.1186/s13643-016-0258-9 (PMC4862155; doi:10.1186/s13643-016-0258-9)
Supplement: Additional file 1: — Search Strategy. Search Strategy and MeSH/keywords used for searching. (DOCX 32 kb) [file 13643_2016_258_MOESM1_ESM.docx]

**Quality of conduct and reporting in rapid reviews: An exploration of compliance with PRISMA and AMSTAR guidelines.** S Kelly et al.

**ADDITIONAL FILE 1: SEARCH STRATEGY**

**MeSH and Keywords**

| **Concept 1 AND** | **Concept 2** | OR |
| --- | --- | --- |
| **MeSH** | | |
| Review literature as topic[mh] | Decision making[mh] OR decisionmaking[tiab] OR “decision maker*”[tiab] |  |
| Meta-analysis as topic[mh] | Decision making, organizational[mh] |  |
| Technology assessment, biomedical[mh] | Decision support techniques[mh] |  |
| Technology assessment, biomedical/methods | Health policy[mh] |  |
| Technology assessment, biomedical/organization & administration | Public policy[mh] |  |
| Technology assessment, biomedical/standards | Policy making[mh] OR policy making[tiab] OR policymaking[tiab] |  |
| Evidence based practice[mh] | Information dissemination[mh] |  |
| Evidence based medicine[mh] |  |  |
|  | Time factors[mh] |  |
| **Non-MeSH** | | |
| HTA[ti] OR HTAs[ti] | Rapid[tiab]  Scoping[tiab] | “rapid review”[tiab] OR “rapid reviews”[tiab] |
| “technology assessment”[ti] | Expedit*[tiab] | “scoping review”[tiab] OR “scoping reviews”[tiab] |
|  | Accelerate*[tiab] | “knowledge synthesis”[tiab] |
|  | Express[tiab] | (knowledge[mh] OR knowledge[ti]) AND (synthesis[ti] OR syntheses[ti] OR summary[ti] OR summaries[ti]) |
|  | “Fast-track”[tiab] OR Fast[tiab] |  |
|  | Quick[tiab] |  |
|  | Turnaround[tiab] |  |
|  | Quick[ti] |  |
|  | Timeline*[ti] |  |
|  | Speedy[ti] |  |
|  | “on demand”[ti] |  |

**MEDLINE** (Ovid MEDLINE(R) In-Process & Other Non-Indexed Citations, Ovid MEDLINE(R) Daily and Ovid MEDLINE(R) 1948 to Present; searched Oct 25, 2011)

| 1 | rapid review*.mp. | 121 |
| --- | --- | --- |
| 2 | scoping review*.mp. | 93 |
| 3 | expedited review*.mp. | 40 |
| 4 | accelerated review*.mp. | 7 |
| 5 | exp "Review Literature as Topic"/ or systematic review.mp. | 32758 |
| 6 | (rapid or faster or accelerate*).ti. | 81318 |
| 7 | 5 and 6 | 109 |
| 8 | knowledge synthesis.mp. | 53 |
| 9 | knowledge syntheses.mp. | 7 |
| 10 | *Knowledge/ | 2531 |
| 11 | knowledge.ti. | 31409 |
| 12 | 10 or 11 | 32663 |
| 13 | (synthes* or summar*).ti. | 222597 |
| 14 | 12 and 13 | 141 |
| 15 | exp Technology Assessment, Biomedical/ | 8548 |
| 16 | exp Policy Making/ | 16205 |
| 17 | exp Decision Making/ | 101899 |
| 18 | 16 or 17 | 116769 |
| 19 | 15 and 18 | 631 |
| 20 | exp "Review Literature as Topic"/ | 5929 |
| 21 | exp Meta-Analysis as Topic/ | 11941 |
| 22 | exp Technology Assessment, Biomedical/ | 8548 |
| 23 | exp Comparative Effectiveness Research/ | 568 |
| 24 | exp Evidence-Based Practice/ | 48686 |
| 25 | exp Evidence-Based Medicine/ | 44873 |
| 26 | (HTA or HTAs or technology assessment* or comparative effectiveness).ti. | 2678 |
| 27 | 20 or 21 or 22 or 23 or 24 or 25 or 26 | 72765 |
| 28 | exp Decision Making/ | 101899 |
| 29 | exp Decision Making, Organizational/ | 10035 |
| 30 | decisionmaking.mp. | 429 |
| 31 | decision making.mp. | 103731 |
| 32 | decision maker*.mp. | 5692 |
| 33 | decisionmaker*.mp. | 172 |
| 34 | exp Decision Support Techniques/ | 50653 |
| 35 | exp Health Policy/ | 70887 |
| 36 | exp Public Policy/ | 96740 |
| 37 | exp Policy Making/ | 16205 |
| 38 | (policy making or policymaking or policy maker* or policymaker*).mp. | 22946 |
| 39 | exp Information Dissemination/ | 7648 |
| 40 | 28 or 29 or 30 or 31 or 32 or 33 or 34 or 35 or 36 or 37 or 38 or 39 | 310710 |
| 41 | 27 and 40 | 10999 |
| 42 | rapid.mp. | 404022 |
| 43 | scoping.mp. | 275 |
| 44 | expedit*.mp. | 9354 |
| 45 | rapid update.mp. | 7 |
| 46 | express.mp. | 123471 |
| 47 | fast-track*.mp. | 1705 |
| 48 | (fast or faster or fasttrack*).mp. | 222850 |
| 49 | (quick or quicker).mp. | 28931 |
| 50 | speedy.mp. | 843 |
| 51 | turnaround.mp. | 2154 |
| 52 | timeline*.mp. | 3277 |
| 53 | on demand.mp. | 3390 |
| 54 | (rigor or rigourous or rigour or rigourous).mp. | 4444 |
| 55 | accelerate*.mp. | 94672 |
| 56 | exp Time Factors/ | 914949 |
| 57 | 42 or 43 or 44 or 45 or 46 or 47 or 48 or 49 or 50 or 51 or 52 or 53 or 54 or 55 or 56 | 1697106 |
| 58 | 41 and 57 | 653 |
| 59 | 1 or 2 or 3 or 4 or 7 or 8 or 9 or 14 or 19 or 58 | 1772 |

**INCLUDED AND EXCLUDED STUDY LISTS**

**Included Unpublished (Grey Literature) Rapid Reviews (n=33)**

1. Self-care support for people with COPD. [Internet] UK. York University, Centre for Reviews and Dissemination/National Institute for Health Research 2014. Available from: https://www.york.ac.uk/media/crd/COPD%20self%20care.pdf.

2. Lead Exposures Among Recreational Shooters. [Internet] Ontario, Canada. Public Health Ontario2014. Available from: http://www.publichealthontario.ca/en/BrowseByTopic/EnvironmentalandOccupationalHealth/Pages/Lead-Exposures-Among-Recreational-Shooters.aspx#.VT_FLdJVhBc.

3. Australian Safety and Efficacy Register of New Interventional Procedures - Surgical (ASERNIP-S). Online programs for weight loss. Brisbane. ASERNIP-S2013. Available from: http://www.health.qld.gov.au/healthpact/docs/briefs/WP176.pdf.

4. Canadian Centre on Substance Abuse (CCSA). Universal, Family-based Substance Abuse Prevention for Youth. [Internet]. CCSA2014. Available from: http://www.ccsa.ca/Resource%20Library/CCSA-Universal-Family-based-Prevention-Youth-Rapid-Review-2014-en.pdf.

5. World Health Organization (WHO). Personal Protective Equipment in the context of filovirus disease outbreak response. [Internet]. World Health Organization (WHO)2014. Available from: http://www.who.int/csr/resources/publications/ebola/ppe-guideline/en/.

6. S Baidoobonso. Effect of supportive interventions on informal caregivers of people at the end of life: A rapid review. Health Quality Ontario2014. Available from: http://www.hqontario.ca/Portals/0/Documents/eds/rapid-reviews/eol-supportive-interventions-1411-en.pdf.

7. N Burrowes. Body image – a rapid evidence assessment of the literature. London, England. United Kingdom Government Equalities Office and the Department for Culture, Media & Sport2013. Available from: https://www.gov.uk/government/publications/body-confidence-a-rapid-evidence-assessment-of-the-literature.

8. CADTH. Sofosbuvir for the Treatment of Patients with Genotype 4 Hepatitis C: A Review of the Clinical Efficacy, Cost-effectiveness, and Guidelines. [Internet]. CADTH2014. Available from: https://www.cadth.ca/sofosbuvir-treatment-patients-genotype-4-hepatitis-c-review-clinical-efficacy-cost-effectiveness-and.

9. Evidence-based Synthesis Program (ESP) Center. Evidence Map of Yoga for High-Impact Conditions Affecting Veterans. [Internet] Washington, USA: Department of Veterans Affairs, Veterans Health Administration, Quality Enhancement Research Initiative, Health Services Research and Development Service; 2014; Available from: http://www.hsrd.research.va.gov/publications/esp/yoga.pdf.

10. M D’Eath, MM Barry, J Sixsmith. A rapid evidence review of health advocacy for communicable diseases. [Internet] Ireland. Health Promotion Research Centre, University of Ireland Galway2014. Available from: http://ecdc.europa.eu/en/publications/Publications/Health-advocacy-technical-report-January-2014.pdf.

11. Scottish Health Technologies Group. Transcathetyer aortic valve implantation (TAVI) for severe symptomatic aortic stenosis in adults who are not eligible for surgery. [Internet] Scotland. Scottish Health Technologies Group2014. Available from: http://www.healthcareimprovementscotland.org/our_work/technologies_and_medicines/shtg_-_evidence_notes/evidence_note_51.aspx.

12. SL Gurgacz, R Lambert, A Vogan, Cooter R, Mutimer K, Maddern G. ASERNIP-S report no. 81 - Breast prosthesis implantation for reconstructive and cosmetic surgery: A rapid review. Adelaide, South Australia. Australian Safety and Efficacy Register of New Interventional Procedures-Surgical (ASERNIP-S)2013. Available from: http://www.surgeons.org/media/20844776/rpt_2014-02-04_rr_breast_implantation__2_.pdf.

13. MM Hirji, MG Wilson, K Yacoub, A Bhuiya. Identifying Suicide Prevention interventions. [Internet]. McMaster Health Forum, McMaster University, Canada2014. Available from: http://www.mcmasterhealthforum.org/docs/default-source/Product-Documents/rapid-responses/identifying-suicide-prevention-interventions.pdf?sfvrsn=2.

14. H Hiscock, P Perera, K McLean, G Roberts. Variation in paediatric clinical practice: An Evidence Check review brokered by the Sax Institute (www.saxinstitute.org.au) for NSW Kids and Families. November 2014. Available from: http://www.saxinstitute.org.au/wp-content/uploads/Report_Variation-in-paediatric-clinical-practice.pdf.

15. ECRI Institute. Automated Technologies for Patient Room Disinfection and Decontamination of Biosafety Level 4 Infectious Agents, Including Ebola Virus. [Internet]. ECRI Institute2014. Available from: https://www.ecri.org/components/Hotline/Pages/14374.aspx?tab=2.

16. Institute of Health Economics. Total prosthetic replacement of the temporomandibular joint: a rapid evidence assessment and economic analysis. Edmonton, Alberta. Institute of Health Economics2013. Available from: http://www.ihe.ca/publications/total-prosthetic-replacement-of-the-temporomandibular-joint-a-rapid-evidence-assessment-and-economic-analysis.

17. N Lapczak, S Hertz. Review of evidence on efective postpartum smoking relapse prevention interventions. [Internet]. Region of Peel, Canada2014. Available from: http://www.peelregion.ca/health/library/pdf/rapid-review-smoking-relapse-prevention.pdf.

18. A Leof, M Thielke, A Gerrity, V King. Sofosbuvir for the Treatment of Hepatitis C and Evaluation of the 2014 American Association for the Study of Liver Diseases Treatment Guidelines. [Internet] USA. Oregon Health and Safety University - Centre for Evidence-based Policy2014. Available from: http://www.ohsu.edu/xd/research/centers-institutes/evidence-based-policy-center/med/upload/Sofosbuvir_for_HepatitisC_FINAL_5_19_2014.pdf.

19. M Masso, C Thompson. Rapid Review of the Nurse Practitioner Literature. [Internet] New South Wales. Government of New South Wales, Australia2014. Available from: http://www.health.nsw.gov.au/nursing/practice/Publications/nurse-practitioner-review.pdf.

20. C Miani, S Ball, E Pitchforth, J Exley, S King, M Roland, J Fuld, E Nolte. Organisational interventions to reduce length of stay in hospital: a rapid evidence assessment. [Internet]. RAND Corporation for the National Institute for Health Research2014. Available from: http://www.journalslibrary.nihr.ac.uk/hsdr/volume-2/issue-52#abstract.

21. Lannin NA, Morarty J, Laver K. Segmented Rehabilitation: A Rapid Review. Melbourne, Australia. Departments of Occupational Therapy, La Trobe University and Alfred Health for The Institute for Safety, Compensation and Recovery Research (ISCRR)2013 Contract No.: ISCRR Research Report # 022.029-R1C. Available from: https://www.tac.vic.gov.au/__data/assets/pdf_file/0016/125404/Segmented-rehabilitation-for-people-after-ABI.pdf.

22. P Navarro, S Bornstein, J O'Loughlin. Strategies for Health promotion: Healthy Dietary Habits. [Internet] Canada. Newfoundland & Labrador Centre for Applied Health Research (NLCAHR), Contextualized Health Research Synthesis Program (CHRSP).2014. Available from: http://www.nlcahr.mun.ca/CHRSP/RER_HEALTH_PROMOTIONS.pdf.

23. Ontario HIV Treatment Network. What is the effectiveness of supervised injections? [Internet]. Ontario HIV Treatment Network2014. Available from: http://www.ohtn.on.ca/Pages/Knowledge-Exchange/Rapid-Responses/Documents/RR83-Supervised-Injection-Effectiveness.pdf.

24. Health Quality Ontario. Vitamin B12 and cognitive function: An Evidence-Based Analysis [Internet]. 2013. Available from: http://www.hqontario.ca/Portals/0/Documents/eds/ohtas/eba-vitamin-b12-cognitive-function-131125-en.pdf.

25. R Parry, J Seymour, B Whittaker, I Bird, C Cox. Rapid evidence review: Pathways focused on the dying phase in end of life care and their key components. United Kingdom. British Department of Health, Sue Ryder Care Centre for the Study of Supportive Palliative and End of Life Care UoN;2013. Available from: http://socialwelfare.bl.uk/subject-areas/services-activity/health-services/departmentofhealth/rapid13.aspx.

26. C Stansfield, N Rumse, J Thomas. Psychosocial predictors, assessment and outcomes of cosmetic interventions: a systematic rapid evidence review. London. EPPI-Centre, Social Science Research Unit, Institute of Education, University of London.2013. Available from: http://eppi.ioe.ac.uk/cms/LinkClick.aspx?fileticket=Ge_RehINz8Q%3D.

27. Syful Azlie Md Fuzi. Ecological Disinfectant (Product name redacted for agency posting). Putrajaya, Malaysia. Health Technology Assessment Section (MaHTAS),Medical Development Division, Ministry of Health Malaysia.2013. Available from: http://www.moh.gov.my/index.php/database_stores/store_view_page/30/211.

28. KS Canada The Knowledge Synthesis (KS) Group, Ottawa Methods Centre, Ottawa Hospital Research Institute. Do Outcomes Vary Among Different Curricula or Models of Delivery for Pre-licensure Nursing Education? A Rapid Review. Ottawa. 2013. Available from: Available on request.

29. Ottawa Methods Centre The Knowledge Synthesis Group and Cochrane Collaborators, Ottawa Hospital Research Institute. Effects of Performing Complex Pediatric Intracavitary (IC) Surgical Procedures in Specialized versus Non-specialized Centers in High Risk Children: Cochrane Response Rapid Review. Ottawa, Ontario, Canada: The Cochrane Collaboration; 2013; Available from: http://innovations.cochrane.org/sites/innovations.cochrane.org/files/CHA-RR%20Evidence%20Summary-Final%2024May2013.pdf.

30. J Turner, L Preston, A Booth, C O’Keeffe, F Campbell, A Jesurasa, K Cooper, E Goyder. What evidence is there for a relationship between organizational features and patient outcomes in congenital heart disease services? A rapid review. [Internet]. School for Health and Related Research (ScHARR), University of Sheffield.2014. Available from: http://www.nets.nihr.ac.uk/__data/assets/pdf_file/0017/118511/HSDR_WR1_13-05-12.pdf.

31. J Ubido, A Scott-Samuel. An evaluation of the cost effectiveness of monitored dosage systems (MDS) as an aid to maintaining independence in taking medication. Liverpool Public Health Observatory2014. Available from: https://www.liv.ac.uk/media/livacuk/instituteofpsychology/publichealthobservatory/LPHO,monitored,dosage,system_,final.pdf.

32. Rapid Review Unit. Comparative safety and effectiveness of inhaled long-acting agents (corticosteroids, beta agonists) for chronic obstructive pulmonary disease (COPD): A rapid review and network meta-analysis. Toronto. Ontario Drug Policy Research Network (ODPRN), St. Michaels Hospital2014. Available from: Available on request.

33. Public Health Wales. Primary Care in Wales: Rapid review of models and policy. [Internet] Wales. Public Health Wales2014. Available from: http://www.wales.nhs.uk/sitesplus/documents/888/Primary%20care%20models%20and%20policies-%20rapid%20review%20SUMMARY%20July%202014%20FINAL.PDF.

**Included journal-published studies (n = 33)**

1. Bungay H, Vella-Burrows T. The effects of participating in creative activities on the health and well-being of children and young people: a rapid review of the literature. Perspectives in public health. 2013 Jan;133(1):44-52.

2. Dennis SM, Harris M, Lloyd J, Powell Davies G, Faruqi N, Zwar N. Do people with existing chronic conditions benefit from telephone coaching? A rapid review. Australian health review : a publication of the Australian Hospital Association. 2013 Jun;37(3):381-8.

3. Jarrett N, Scott I, Addington-Hall J, Amir Z, Brearley S, Hodges L, et al. Informing future research priorities into the psychological and social problems faced by cancer survivors: a rapid review and synthesis of the literature. European journal of oncology nursing : the official journal of European Oncology Nursing Society. 2013 Oct;17(5):510-20.

4. Kim DG, Choi YY, An JY, Kwon IG, Cho I, Kim YM, et al. Comparing the short-term outcomes of totally intracorporeal gastroduodenostomy with extracorporeal gastroduodenostomy after laparoscopic distal gastrectomy for gastric cancer: a single surgeon's experience and a rapid systematic review with meta-analysis. Surgical endoscopy. 2013 Sep;27(9):3153-61.

5. McLean SL, Blenkinsopp A, Bennett MI. Using haloperidol as an antiemetic in palliative care: informing practice through evidence from cancer treatment and postoperative contexts. Journal of pain & palliative care pharmacotherapy. 2013 Jun;27(2):132-5.

6. Ndumbe-Eyoh S, Moffatt H. Intersectoral action for health equity: a rapid systematic review. BMC Public Health. 2013;13:1056.

7. Trivedy CR, Cooke MW. Unscheduled return visits (URV) in adults to the emergency department (ED): a rapid evidence assessment policy review. Emergency medicine journal : EMJ. 2013 Oct 28.

8. Zeno SA, Purvis D, Crawford C, Lee C, Lisman P, Deuster PA. Warm-ups for military fitness testing: rapid evidence assessment of the literature. Medicine and science in sports and exercise. 2013 Jul;45(7):1369-76.

9. Banbury A, Roots A, Nancarrow S. Rapid review of applications of e-health and remote monitoring for rural residents. The Australian journal of rural health. 2014 Oct;22(5):211-22.

10. Beall RF, Baskerville N, Golfam M, Saeed S, Little J. Modes of delivery in preventive intervention studies: a rapid review. European journal of clinical investigation. 2014 Jul;44(7):688-96.

11. Brunton G, Paraskeva N, Caird J, Bird KS, Kavanagh J, Kwan I, et al. Psychosocial predictors, assessment, and outcomes of cosmetic procedures: a systematic rapid evidence assessment. Aesthetic plastic surgery. 2014 Oct;38(5):1030-40.

12. Chaiyachati KH, Ogbuoji O, Price M, Suthar AB, Negussie EK, Barnighausen T. Interventions to improve adherence to antiretroviral therapy: a rapid systematic review. AIDS (London, England). 2014 Mar;28 Suppl 2:S187-204.

13. Costello RB, Lentino CV, Boyd CC, O'Connell ML, Crawford CC, Sprengel ML, et al. The effectiveness of melatonin for promoting healthy sleep: a rapid evidence assessment of the literature. Nutrition journal. 2014;13:106.

14. Delgado R, York A, Lee C, Crawford C, Buckenmaier C, 3rd, Schoomaker E, et al. Assessing the quality, efficacy, and effectiveness of the current evidence base of active self-care complementary and integrative medicine therapies for the management of chronic pain: a rapid evidence assessment of the literature. Pain medicine (Malden, Mass). 2014 Apr;15 Suppl 1:S9-20.

15. Dorresteijn PM, Ipenburg NA, Murphy KJ, Smit M, van Vulpen JK, Wegner I, et al. Rapid Systematic Review of Normal Audiometry Results as a Predictor for Benign Paroxysmal Positional Vertigo. Otolaryngology--head and neck surgery : official journal of American Academy of Otolaryngology-Head and Neck Surgery. 2014 Mar 18;150(6):919-24.

16. Garrett B, Taverner T, Masinde W, Gromala D, Shaw C, Negraeff M. A rapid evidence assessment of immersive virtual reality as an adjunct therapy in acute pain management in clinical practice. The Clinical journal of pain. 2014 Dec;30(12):1089-98.

17. Hithersay R, Strydom A, Moulster G, Buszewicz M. Carer-led health interventions to monitor, promote and improve the health of adults with intellectual disabilities in the community: a systematic review. Research in developmental disabilities. 2014 Apr;35(4):887-907.

18. Khorsan R, Crawford C, Ives JA, Walter AR, Jonas WB. The effect of omega-3 fatty acids on biomarkers of inflammation: a rapid evidence assessment of the literature. Military medicine. 2014 Nov;179(11 Suppl):2-60.

19. Lal S, Adair CE. E-mental health: a rapid review of the literature. Psychiatric services (Washington, DC). 2014 Jan 1;65(1):24-32.

20. Loveday HP, Wilson JA, Kerr K, Pitchers R, Walker JT, Browne J. Association between healthcare water systems and Pseudomonas aeruginosa infections: a rapid systematic review. The Journal of hospital infection. 2014 Jan;86(1):7-15.

21. Luckett T, Phillips J, Agar M, Virdun C, Green A, Davidson PM. Elements of effective palliative care models: a rapid review. BMC Health Serv Res. 2014;14:136.

22. Menear M, Briand C. Implementing a continuum of evidence-based psychosocial interventions for people with severe mental illness: part 1-review of major initiatives and implementation strategies. Canadian journal of psychiatry Revue canadienne de psychiatrie. 2014 Apr;59(4):178-86.

23. Moe-Byrne T, Chambers D, Harden M, McDaid C. Behaviour change interventions to promote prescribing of generic drugs: a rapid evidence synthesis and systematic review. BMJ open. 2014;4(5):e004623.

24. Paton F, Chambers D, Wilson P, Eastwood A, Craig D, Fox D, et al. Effectiveness and implementation of enhanced recovery after surgery programmes: a rapid evidence synthesis. BMJ open. 2014;4(7):e005015.

25. Reinink H, Wegner I, Stegeman I, Grolman W. Rapid systematic review of repeated application of the epley maneuver for treating posterior BPPV. Otolaryngology--head and neck surgery : official journal of American Academy of Otolaryngology-Head and Neck Surgery. 2014 Sep;151(3):399-406.

26. Saeed S, Golfam M, Beall RF, Ashbury FD, Palmer LJ, Little J. Effectiveness of individual-focused interventions to prevent chronic disease. European journal of clinical investigation. 2014 Sep;44(9):883-91.

27. Treanor CJ, Donnelly M. The late effects of cancer and cancer treatment: a rapid review. The Journal of community and supportive oncology. 2014 Apr;12(4):137-48.

28. van der Scheer-Horst ES, van Benthem PP, Bruintjes TD, van Leeuwen RB, van der Zaag-Loonen HJ. The efficacy of vestibular rehabilitation in patients with benign paroxysmal positional vertigo: a rapid review. Otolaryngology--head and neck surgery : official journal of American Academy of Otolaryngology-Head and Neck Surgery. 2014 Nov;151(5):740-5.

29. van der Veen EL, van Hulst RA, de Ru JA. Hyperbaric Oxygen Therapy in Acute Acoustic Trauma: A Rapid Systematic Review. Otolaryngology--head and neck surgery : official journal of American Academy of Otolaryngology-Head and Neck Surgery. 2014 Mar 19;151(1):42-5.

30. van Duijn JG, Isfordink LM, Nij Bijvank JA, Stapper CW, van Vuren AJ, Wegner I, et al. Rapid Systematic Review of the Epley Maneuver for Treating Posterior Canal Benign Paroxysmal Positional Vertigo. Otolaryngology--head and neck surgery : official journal of American Academy of Otolaryngology-Head and Neck Surgery. 2014 Mar 31;150(6):925-32.

31. Wegner I, Niesten ME, van Werkhoven CH, Grolman W. Rapid Systematic Review of the Epley Maneuver versus Vestibular Rehabilitation for Benign Paroxysmal Positional Vertigo. Otolaryngology--head and neck surgery : official journal of American Academy of Otolaryngology-Head and Neck Surgery. 2014 May 20;151(2):201-7.

32. Wolfenden L, Carruthers J, Wyse R, Yoong S. Translation of tobacco control programs in schools: findings from a rapid review of systematic reviews of implementation and dissemination interventions. Health promotion journal of Australia : official journal of Australian Association of Health Promotion Professionals. 2014 Aug;25(2):136-8.

33. Cooper CL, Hind D, Duncan R, Walters S, Lartey A, Lee E, et al. A rapid review indicated higher recruitment rates in treatment trials than in prevention trials. J Clin Epidemiol. 2015 Mar;68(3):347-54.

**Excluded journal-published studies (n = 77)**

1. Amyot R. [Rapid review of the events leading to the establishment of the Conseil Medical du Canada granting the federal license]. L'union medicale du Canada. 1960 May;89:633-5.

2. Attree P, French B, Milton B, Povall S, Whitehead M, Popay J. The experience of community engagement for individuals: a rapid review of evidence. Health Soc Care Community. 2011 May;19(3):250-60.

3. Baker A, Melcher D, Smith R. Rapid review. Cytopathology : official journal of the British Society for Clinical Cytology. 1996 Aug;7(4):294-5.

4. Bambra C, Joyce KE, Bellis MA, Greatley A, Greengross S, Hughes S, et al. Reducing health inequalities in priority public health conditions: using rapid review to develop proposals for evidence-based policy. J Public Health (Oxf). 2010 Dec;32(4):496-505.

5. Beiri A, Alani A, Ibrahim T, Taylor GJ. Trauma rapid review process: efficient out-patient fracture management. Annals of the Royal College of Surgeons of England. 2006 Jul;88(4):408-11.

6. Blackwell E, McKinney WM. A new system for rapid review of clinical studies and more efficient group teaching in ultrasound. Journal of clinical ultrasound : JCU. 1975 Mar;3(1):55-6.

7. Boycott N, Schneider J, McMurran M. Additional interventions to enhance the effectiveness of individual placement and support: a rapid evidence assessment. Rehabilitation research and practice. 2012;2012:382420.

8. Brearley SG, Stamataki Z, Addington-Hall J, Foster C, Hodges L, Jarrett N, et al. The physical and practical problems experienced by cancer survivors: a rapid review and synthesis of the literature. EurJ OncolNurs. 2011;15(3):204-12.

9. Bulgaresi P, Cariaggi MP, Troni GM, Ciatto S. Quality control of the autopap screening system employed as a primary screening device: rapid review of smears coded as no further review. Tumori. 2006 Jul-Aug;92(4):276-8.

10. Bury M, Newbould J, Taylor D. A rapid review of the current state of knowledge regarding lay-led self-management of chronic illness. Evidence review. London: National Institute for Health and Clinical Excellence (NICE); 2005.

11. Casadesus D. Surgical resection of rectal adenoma: a rapid review. World J Gastroenterol. 2009;15(31):3851-4.

12. Chrome P. Rapid review of medicine in old age. GM: Midlife & Beyond. 2007;40(2):92-.

13. Curson JA, Dell ME, Wilson RA, Bosworth DL, Baldauf B. Who does workforce planning well? Workforce review team rapid review summary. International journal of health care quality assurance. 2010;23(1):110-9.

14. Faraker CA. Rapid review. Cytopathology : official journal of the British Society for Clinical Cytology. 1998 Apr;9(2):71-6.

15. Faraker CA. Invited commentary--Rapid review: current practice. Cytopathology : official journal of the British Society for Clinical Cytology. 2001 Aug;12(4):249-50.

16. Faraker CA, Boxer ME. Rapid review (partial rescreening) of cervical cytology. Four years experience and quality assurance implications. Journal of clinical pathology. 1996 Jul;49(7):587-91.

17. Fitzpatrick-Lewis D, Ganann R, Krishnaratne S, Ciliska D, Kouyoumdjian F, Hwang SW. Effectiveness of interventions to improve the health and housing status of homeless people: a rapid systematic review. BMC Public Health. 2011;11:638.

18. Fontanarosa PB, DeAngelis CD. Update on JAMA-EXPRESS: rapid review and publication. Jama. 2008 Dec 24;300(24):2920-1.

19. Foxcroft DR, Milne R. Orlistat for the treatment of obesity: rapid review and cost-effectiveness model. Obesity reviews : an official journal of the International Association for the Study of Obesity. 2000 Oct;1(2):121-6.

20. Frist S. Rapid review of cervical cytology. Journal of clinical pathology. 1997 Jan;50(1):87.

21. Geddes R, Frank J, Haw S. A rapid review of key strategies to improve the cognitive and social development of children in Scotland. Health Policy. 2011 Jun;101(1):20-8.

22. Golfam M, Beall R, Brehaut J, Saeed S, Relton C, Ashbury FD, et al. Comparing alternative design options for chronic disease prevention interventions. European journal of clinical investigation. 2015 Jan;45(1):87-99.

23. Greenwald JL, Burstein GR, Pincus J, Branson B. A rapid review of rapid HIV antibody tests. Current infectious disease reports. 2006 Mar;8(2):125-31.

24. Gush C, Borriello P. An overview of the UK Department of Health's Rapid Review Panel. The Journal of hospital infection. 2007 Jun;65 Suppl 2:27-9.

25. Henderson S, Stevens M, Walker T. Rapid review of liquid-based smears as a quality control measure. Diagnostic cytopathology. 2004 Sep;31(3):141-6.

26. Hildon Z, Neuburger J, Allwood D, van der Meulen J, Black N. Clinicians' and patients' views of metrics of change derived from patient reported outcome measures (PROMs) for comparing providers' performance of surgery. BMC Health Serv Res. 2012;12:171.

27. Hill R, Bagust A, Bakhai A, Dickson R, Dundar Y, Haycox A, et al. Coronary artery stents: a rapid systematic review and economic evaluation. Health Technol Assess. 2004 Sep;8(35):iii-iv, 1-242.

28. Jahangirian M, Eldabi T, Garg L, Jun GT, Naseer A, Patel B, et al. A rapid review method for extremely large corpora of literature: Applications to the domains of modelling, simulation, and management. International Journal of Information Management. 2011;31(3):234-43.

29. James A, McNamee D, Horton R. Rapid reviews in The Lancet--and beyond. Lancet. 2002;360(9327):102.

30. Kelley LS. What is in store for 2004? Rapid review will mean cutting edge information to you! Journal of gerontological nursing. 2003 Dec;29(12):5.

31. Kelly BJ, Perkins DA, Fuller JD, Parker SM. Shared care in mental illness: A rapid review to inform implementation. Int J MentHealth Syst. 2011;5(1):31.

32. Konnyu KJ, Kwok E, Skidmore B, Moher D. The effectiveness and safety of emergency department short stay units: a rapid review. Open Med. 2012;6(1):e10-6.

33. Lee BC, Lam SY, Walker T. Comparison of false negative rates between 100% rapid review and 10% random full rescreening as internal quality control methods in cervical cytology screening. Acta cytologica. 2009 May-Jun;53(3):271-6.

34. Manrique EJ, Souza NL, Tavares SB, Albuquerque ZB, Zeferino LC, Amaral RG. Analysis of the performance of 100% rapid review using an average time of 1 and 2 minutes according to the quality of cervical cytology specimens. Cytopathology : official journal of the British Society for Clinical Cytology. 2011 Jun;22(3):195-201.

35. McMurran M. Individual-level interventions for alcohol-related violence: a rapid evidence assessment. Criminal behaviour and mental health : CBMH. 2012 Feb;22(1):14-28.

36. Moyad MA. The use of complementary/preventive medicine to prevent prostate cancer recurrence/progression following definitive therapy. Part II--rapid review of dietary supplements. Current opinion in urology. 2003 Mar;13(2):147-51.

37. Moyad MA. Fad diets and obesity--Part III: a rapid review of some of the more popular low-carbohydrate diets. Urologic nursing. 2004 Oct;24(5):442-5.

38. Moyad MA. Vitamin D: a rapid review. Urologic nursing. 2008 Oct;28(5):343-9, 84; quiz 50.

39. Moyad MA. Vitamin D: a rapid review: the "ideal" vitamin D blood level. Dermatology Nursing. 2009;21(1).

40. Moyad MA. Vitamin D: a rapid review. Dermatology nursing / Dermatology Nurses' Association. 2009 Jan-Feb;21(1):25-30, 55.

41. Moyad MA. Heart health = urologic health and heart unhealthy = urologic unhealthy: rapid review of lifestyle changes and dietary supplements. UrolClin North Am. 2011;38(3):359-67.

42. Moyad MA, Merrick GS. Cholesterol, cholesterol-lowering agents/statins, and urologic disease: Part III--A rapid review of FDA-approved cholesterol-lowering agents. Urologic nursing. 2006 Aug;26(4):330-2.

43. O'Sullivan JP, Chapman PA, Jenkins L, Smith R. Variables involved in rapid review. Cytopathology : official journal of the British Society for Clinical Cytology. 1999 Apr;10(2):144-5.

44. Parker S, Fuller J. Are nurses well placed as care co-ordinators in primary care and what is needed to develop their role: a rapid review? Health Soc Care Community. 2015 Feb 9.

45. Revere D, Fuller S, Bugni PF, Martin GM. An information extraction and representation system for rapid review of the biomedical literature. Stud Health Technol Inform. 2004;107(Pt 2):788-92.

46. Secrist J. Osteoporosis. Part IV -- rapid review of drug therapies (A to Z) for preventing male osteoporosis/fractures. Urologic nursing. 2003 Apr;23(2):168-74.

47. Slater DN. Sensitivity of primary screening by rapid review: 'to act or not to act on the results, that is the question'. Cytopathology : official journal of the British Society for Clinical Cytology. 1998 Apr;9(2):77-83.

48. Stengel D, Ekkernkamp A, Dettori J, Hanson B, Sturmer KM, Siebert H. [A rapid review of the minimum quality problems using total knee arthroplasty as an example. Where do the magical threshold values come from?]. Unfallchirurg. 2004 Oct;107(10):967-88.

49. Sutton A, Grant MJ. Cost-effective ways of delivering enquiry services: a rapid review. Health Info Libr J. 2011;28(4):249-55.

50. Szmuda T, Sloniewski P, Waszak PM, Springer J, Szmuda M. Towards a new treatment paradigm for ruptured blood blister-like aneurysms of the internal carotid artery? A rapid systematic review. 2015 Mar 19.

51. Tarducci M. [Old and new themes (projected toward a more ample delay of international approaches) in evaluations of ocular damage in accident patients: rapid review of eminently practical nature]. Bollettino d'oculistica. 1960 Dec;39:903-22.

52. Tavares SB, Alves de Sousa NL, Manrique EJ, Pinheiro de Albuquerque ZB, Zeferino LC, Amaral RG. Improvement in the routine screening of cervical smears: A study using rapid prescreening and 100% rapid review as internal quality control methods. Cancer cytopathology. 2011 Dec 25;119(6):367-76.

53. Toomey E, Currie-Murphy L, Matthews J, Hurley DA. The effectiveness of physiotherapist-delivered group education and exercise interventions to promote self-management for people with osteoarthritis and chronic low back pain: A rapid review Part I. Manual therapy. 2015 Apr;20(2):265-86.

54. Van de Velde S, De Buck E, Dieltjens T, Aertgeerts B. Medicinal use of potato-derived products: conclusions of a rapid versus full systematic review. Phytotherapy research : PTR. 2011 May;25(5):787-8.

55. Wilson NJ, Molyneux AJ. Rapid review in cervical cytology: a retrospective review of cases detected on rapid review within a DGH cytology department and subsequent outcome. Cytopathology : official journal of the British Society for Clinical Cytology. 2004 Apr;15(2):93-6.

56. Zechmeister I, Schumacher I. The impact of health technology assessment reports on decision making in Austria. Int J Technol Assess Health Care. 2012 Jan;28(1):77-84.

57. Ziegler S, Luhmann D, Raspe H, Windeler J. [Rapid reviews for evidence-based decision support. (Restricting) requirements]. ZArztlFortbildQualitatssich. 2001;95(2):105-11.
